# Supplementary material for: A systematic evaluation of in-context learning in large language models for antibody characterization
Source: Genome Biol. 2026 Aug 13;27:352. doi: 10.1186/s13059-026-04240-6 (PMC13757043; doi:10.1186/s13059-026-04240-6)
Supplement: Supplementary file 2 [file 13059_2026_4240_MOESM2_ESM.pdf]

Few-shot (2-shot), Similar (Balanced, Positive-Negative)

Scenario 1b (Binary): Antibody species-origin (Rhesus vs. Human) prediction

You are an AI model specializing in immunology. Your task is to **predict the humanness of antibody** using only the amino acid sequences provided. Recent research has suggested that **the humanness of antibody** can be predicted with antibody sequence alone, without any structural information.
Please strictly follow the format, no other information can be provided. Given the amino acid sequences of an antibody, predict **the humanness of antibody based on its sequence, by analyzing whether it can Human(1) or Rhesus(0).** Consider factors such as physiochemical property, PSSM and amino acid frequency to assess **the humanness of antibody**.
Please answer with *only* 1 or 0 only for the last example. A few examples are provided in the beginning.
Antibody:
QVQLQESGPGLLKPSETLSLTCSVSGGSITSSTYSWGWIRQPPGKGLEWIGSVFYNGDTYYNPSLKSRVTVSVDTSKNQVSLKLSSVTAADTAVYYCTRASIFG
VALDAFDLWGQGTMVSVSS
Humanness: 1
Antibody:
DVQLVESGGGLVQPGGSLRLSCAASGFTFSSYGMSWVRQAPGKGLDWVSYISNVGGITYYADSVKGRFTISRDNSKNTLSLQMNSLRAEDTAVYYCAKGRYY
YDSGFYKPFDYWGQGVLVTVSS
Humanness: 0

Now, predict **the humanness of antibody** for the following antibody sequence based on the criteria above:
Antibody:
QVQLQQWGEGLVKPSETLSLTCAVYGGSVSGYWWGWIRPPPGKGLEWIGRIRSGGSTNDTPSPKSRVTISIDTSKNQFSLKLSSVTAADTAVYYCARLPTAAAL
DYWGQGVLVTVSS
Humanness:

Scenario 2 (Binary): Antibody specificity prediction

You are an AI model specializing in immunology. Your task is to **predict the binding specificity of antibody to the SARS-CoV-2 spike protein** using only the amino acid sequences provided. Recent research has suggested that **the specificity to antigens of antibody** can be predicted with antibody sequence alone, without any structural information.
Please strictly follow the format, no other information can be provided. Given the amino acid sequences of an antibody, predict **the receptor binding specificity of antibody based on its sequence, by analyzing whether it can bind(1) or cannot bind(0).** Consider factors such as physiochemical property, PSSM and amino acid frequency to assess **the binding of antibody.**
Please answer with *only* 1 or 0 only for the last example. A few examples are provided in the beginning.
Antibody:
QVQLVQSGAEVKKPGASVKVSCKASGYTFTSYGISWLRQAPGQGLEWMGWISAYNGDTNYAPKLQGKVTMTTDTSTSTAYMELRSLRSDDTAVYYCARD
LPKISIFGVADYWGQGTLVTVSSDIQMTQSPSSLSASVGDRVTITCRASQSISRYLNWYQQKPGKAPKLLIQAASSLQSGVPSRFSGSGSGTDFTLTISSLQP
EDFATYYCQQSYSTPFTFGPGTKVDIK
Binding: 1
Antibody:
EVQLVESGGGLVQPGGSLRLSCAASGFTFSSYWMHWVRQAPGKGLVWVSRINSDGNSTSYADSVKGRFTISRDNAKNTLYLQMNSLRAEDTAVYYCARD
LPPYYDFWSGLERGMDVWGQGTTVTVSSQSVLTQPPSASGTPGQRVTISCSGSSSNIGSNYVYWYQQLPGTAPKLLIYRNNQRPSGVPDRFSGSKSGTSA
SLAISGLRSEDEADYYCAAWDDSLSGYVFGTGTKVTVL
Binding: 0

Now, predict **the receptor binding specificity** for the following antibody sequence based on the criteria above:
Antibody:
QVQLQESGPGLVKPSETLSLTCTVSGYSISSGYYWGWIRQPPGKGLEWIGSIYHCGSTYYNPSLKSRLTISVDTSKNQFSLKLSSVTAADTAVYYCAREPDY
SDSGGYYSLYYFAYWGQGTLVTVSSSHERTQPPSVSVSPGQTARITCSGDALPKQYAYWYQQKPGQAPVVVIYKDTERPSGIPERISGSSSGTTVTLTISGV
QAEDEADYYCQSADSSGTYVVFGGGTKLTVL
Binding:

Few-shot (4-shot), Similar (Balanced, Positive-Negative)

Scenario 3 (Multiclass): Antibody isotype class prediction

You are an AI model specializing in immunology. Your task is to **predict the heavy chain isotype** using only the amino acid sequences provided. Recent research has suggested that **the heavy chain isotype of antibody** can be predicted with antibody sequence alone, without any structural information.
Please strictly follow the format, no other information can be provided. Given the amino acid sequences of an antibody, predict **the heavy chain isotype of antibody based on its sequence, by analyzing whether it is IGHA (0), IGHD (1), IGHG (2), IGHM (3)**. Consider factors such as physiochemical property, PSSM and amino acid frequency to assess **the heavy chain isotype.**
Please answer with *only* 0, 1, 2 or 3 for the last example. A few examples are provided in the beginning.
Antibody:
QVQLVQSGAEVKKPGASVKVSCKVSGYTLTDLSLHWVRQAPGKGLEWMGGFDPEDEDTIYAQNFKGRLTMTEDTSTDTAYMELSSLRSEDTAVYYCATNQP
TGWELLGDHAFDIWGQGTVVTVSS
Heavy chain isotype: 0
Antibody:
EVQLLESGGGLVQPGGSLRLSCAASGFTFSSYAMSWVRQAPGKGLEWVSAISGSGGSTYYADSVKGRFTISRDNSKNTLYLQMNSLRAEDTAVYYCAKDDSS
GYPNWFDPWGQGTLVTVSS
Heavy chain isotype: 1
Antibody:
EVQLVESGGGLVQPGGSLRVSCAASGFTFSNYAMHWVRQAPGKGLEYISAITSKGAKSFYANSVTGRFNISRDNSKNTLYLQMGSLRVEDTAIYYCVRGDDGT
GYWDYFDYWGQGTLVTVSS
Heavy chain isotype: 2
Antibody:
QVQLVESGGGVVQPGRSLRLSCAASGFTFSSYAMHWVRQAPGKGLEWVAVISYDGSNKYYADSVKGRFTISRDNSKNTLYLQMNSLRAEDTAVYYCARDLR
QLERFVSGNLDYWGQGTLVTVSS
Heavy chain isotype 3

:

Now, predict **the heavy chain isotype** for the following antibody sequence based on the criteria above:
Antibody:
EVQLVESGGGLVQPGGSLRLSCAASGFTFSSYWMSWVRQAPGKGLEWVANIKQDGSEKYYVDSVKGRFTISRDNAKNSLYLQMNSLRAEDTAVYYCARPAT
PTYNWNDAGWFDPWGQGTLVTVSS
Heavy chain isotype:

Fig. S1: Examples of few-shot prompts in three antibody characterization scenarios. The text highlighted in this figure shows the variable inputs specific to each scenario. All other text remains constant across the examples.

Zero-shot

Scenario 1b (Binary): Antibody species-origin (Rhesus vs. Human) prediction

You are an AI model specializing in immunology. Your task is to **predict the humanness of antibody** using only the amino acid sequences provided. Recent research has suggested that **the humanness of antibody** can be predicted with antibody sequence alone, without any structural information.

Please strictly follow the format, no other information can be provided. Given the amino acid sequences of an antibody, predict **the humanness of antibody based on its sequence, by analyzing whether it can Human(1) or Rhesus(0)**. Consider factors such as physiochemical property, PSSM and amino acid frequency to assess **the humanness of antibody.**

Please answer with *only* 1 or 0 only for the last example.

Antibody:

QVQLQQWGEGLVKPSETLSLTCAVYGGSVSGYWWGWIRPPPGKGLEWIGRIRSGGSTNDTPSPKSRVTISIDTSKNQFSLKLSSVTAADTAVYYCARLPTAAA
LDYWGQGVLVTVSS

Humanness:

Scenario 2 (Binary): Antibody specificity prediction

You are an AI model specializing in immunology. Your task is to **predict the binding specificity of antibody to the SARS-CoV-2 spike protein** using only the amino acid sequences provided. Recent research has suggested that **the specificity to antigens of antibody** can be predicted with antibody sequence alone, without any structural information.

Please strictly follow the format, no other information can be provided. Given the amino acid sequences of an antibody, predict **the receptor binding specificity of antibody based on its sequence, by analyzing whether it can bind(1) or cannot bind(0).** Consider factors such as physiochemical property, PSSM and amino acid frequency to assess **the binding of antibody.**

Please answer with *only* 1 or 0 only for the last example.

Antibody:

QVQLQESGPGLVKPSETLSLTCTVSGYSISSGYYWGWIRQPPGKGLEWIGSIYHCGSTYYNPSLKSRLTISVDTSKNQFSLKLSSVTAADTAVYYCAREPDYSD
SGGYYSLYYFAYWGQGTLVTVSSSHERTQPPSVSVSPGQTARITCSGDALPKQYAYWYQQKPGQAPVVVIYKDTERPSGIPERISGSSSGTTVTLTISGVQAED
EADYYCQSADSSGTYVVFGGGTKLTVL

Binding:

Scenario 3 (Multiclass): Antibody isotype class prediction

You are an AI model specializing in immunology. Your task is to **predict the heavy chain isotype** using only the amino acid sequences provided. Recent research has suggested that **the heavy chain isotype of antibody** can be predicted with antibody sequence alone, without any structural information.

Please strictly follow the format, no other information can be provided. Given the amino acid sequences of an antibody, predict **the heavy chain isotype of antibody based on its sequence, by analyzing whether it is IGHA (0), IGHD (1), IGHG (2), IGHM (3).** Consider factors such as physiochemical property, PSSM and amino acid frequency to assess **the heavy chain isotype.**

Please answer with *only* 0, 1, 2 or 3 for the last example.

Antibody:

EVQLVESGGGLVQPGGSLRLSCAASGFTFSSYWMSWVRQAPGKGLEWVANIKQDGSEKYYVDSVKGRFTISRDNAKNSLYLQMNSLRAEDTAVYYCARPAT
PTYNWNDAGWFDPWGQGTLVTVSS

Heavy chain isotype:

Fig. S2: Examples of zero-shot prompts in three antibody characterization scenarios. The text highlighted in this figure shows the variable inputs specific to each scenario. All other text remains constant across the examples.

Few-shot Chain-of-Thought (CoT) (32-shot), Similar (Balanced, Random)

Scenario 1b (Binary): Antibody species-origin (Rhesus vs. Human) prediction

You are an AI model specializing in immunology. Your task is **to predict the humanness of antibody** using only the amino acid sequences provided. Recent research has suggested that **the humanness of antibody** can be predicted with antibody sequence alone, without any structural information.
Please strictly follow the format, no other information can be provided. Given the amino acid sequences of an antibody, predict **the humanness of antibody based on its sequence, by analyzing whether it can Human(1) or Rhesus(0).** Consider factors such as physiochemical property, PSSM and amino acid frequency to assess **the humanness of antibody.**
Please answer with *only* 1 or 0 only for the last example. A few examples are provided in the beginning.
Antibody:
QVQLQQWGEGLVKPSETLSLTCAVYGGSVSGYWWGWIRQPPGKGRGWFGRIRSGGRTNSNPSLKSRVTSSIDTSKNQFSLKLSSVTAADTAVYYCARPTGN
TVTYWGQGVLVTVSS
Humanness: 0
….
Antibody:
QVQLQQWGAGLLKPSETLSLTCAVYGGSFSGYYWSWIRQPPGKGLEWIGEINHSGSTNYNPSLKSRVTISVDTSKNQFSLKLSSVTAADTAVYYCARGGGKAG
WFDPWGQGTLVTVSS
Humanness: 1
Let's solve this problem by splitting it into steps.
Step 1: Please calculate the frequency of each amino acid in the sequence.
Step 2: Please consider the physiochemical property of each amino acid in the sequence.
Now, predict **the humanness of antibody** for the following antibody sequence based on the criteria above:
Antibody:
QVQLQQWGEGLVKPSETLSLTCAVYGGSVSGYWWGWIRPPPGKGLEWIGRIRSGGSTNDTPSPKSRVTISIDTSKNQFSLKLSSVTAADTAVYYCARLPTAAAL
DYWGQGVLVTVSS
Humanness:

Scenario 2 (Binary): Antibody specificity prediction

You are an AI model specializing in immunology. Your task is **to predict the binding specificity of antibody to the SARS-CoV-2 spike protein** using only the amino acid sequences provided. Recent research has suggested that **the specificity to antigens of antibody** can be predicted with antibody sequence alone, without any structural information.
Please strictly follow the format, no other information can be provided. Given the amino acid sequences of an antibody, predict **the receptor binding specificity of antibody based on its sequence, by analyzing whether it can bind(1) or cannot bind(0).** Consider factors such as physiochemical property, PSSM and amino acid frequency to assess **the binding of antibody.**
Please answer with *only* 1 or 0 only for the last example. A few examples are provided in the beginning.
Antibody:
QLQLQESGPGLVKPSETLSLTCTVSGGSISSSSYYWGWIRQPPGKGLEWIGSIYYSGSTYYNPSLKSRVTISVDTSKNQFSLKLSSVTAADTAVYYCARRVITIFG
GAGFDYWGQGTLVTVSSSYELTQPPSVSVSPGQTARITCSADALPKQYAYWYQQKPGQAPVLVIYKDSERPSGIPERFSGSSSGTTVTLTISGVQAEDEADYYC
QSADSSGTYVVFGGGTKLTVL
Binding: 0
…
Antibody:
QVPLQESGPGLVKPSETLSLTCTVSGGSINSYYWNWIRQAPGKGLEWIGNIYYSGSTSYNPSLKSRVTISVDTSKNQFSLKLTSVTAADTAVYYCARGGFYDPYY
FDSWGQGTLVTVSSSFELTQPPSVSVSPGQTARITCSANALPMQYAYWYQQKPGQAPVLVIYKDIERASGIPERFSGSSSGATVTLTISGVQAEDEADYYCQSP
DSSGSYVVFGGGTKVTVL
Binding: 1
Let's solve this problem by splitting it into steps.
Step 1: Please calculate the frequency of each amino acid in the sequence.
Step 2: Please consider the physiochemical property of each amino acid in the sequence.
Now, predict **the receptor binding specificity** for the following antibody sequence based on the criteria above:
Antibody:
QVQLQESGPGLVKPSETLSLTCTVSGYSISSGYYWGWIRQPPGKGLEWIGSIYHCGSTYYNPSLKSRLTISVDTSKNQFSLKLSSVTAADTAVYYCAREPDYSDS
GGYYSLYYFAYWGQGTLVTVSSSHERTQPPSVSVSPGQTARITCSGDALPKQYAYWYQQKPGQAPVVVIYKDTERPSGIPERISGSSSGTTVTLTISGVQAEDE
ADYYCQSADSSGTYVVFGGGTKLTVL
Binding:

Scenario 3 (Multiclass): Antibody isotype class prediction

You are an AI model specializing in immunology. Your task is **to predict the heavy chain isotype** using only the amino acid sequences provided. Recent research has suggested that **the heavy chain isotype of antibody** can be predicted with antibody sequence alone, without any structural information.
Please strictly follow the format, no other information can be provided. Given the amino acid sequences of an antibody, predict **the heavy chain isotype of antibody based on its sequence, by analyzing whether it is IGHA (0), IGHD (1), IGHG (2), IGHM (3).** Consider factors such as physiochemical property, PSSM and amino acid frequency to assess **the heavy chain isotype**.
Please answer with *only* 0, 1, 2 or 3 for the last example. A few examples are provided in the beginning.
Antibody:
EVQLVESGGGLVQPGGSLRLSCAASGFTFSSYWMSWVRQAPGKGLEWVANIKQDGSEKYYVDSVKGRFTISRDNAKNSLYLQMNSLRAEDTAVYYCAGGGY
NWNRAWFDPWGQGTLVTVSS
Heavy chain isotype: 3
…
Antibody:
EVQLVESGGGLVQPGGSLRLSCAASGFTFSSYWMSWVRQAPGKGLEWVANIKQDGSEKYYVDSVKGRFTISRDNAKNSLYLQMNSLRAEDTAVYYCARARS
DYADYVYFDYWGQGTLVTVSS
Heavy chain isotype: 0
Let's solve this problem by splitting it into steps.
Step 1: Please calculate the frequency of each amino acid in the sequence.
Step 2: Please consider the physiochemical property of each amino acid in the sequence.
Now, predict **the heavy chain isotype** for the following antibody sequence based on the criteria above:
Antibody:
EVQLVESGGGLVQPGGSLRLSCAASGFTFSSYWMSWVRQAPGKGLEWVANIKQDGSEKYYVDSVKGRFTISRDNAKNSLYLQMNSLRAEDTAVYYCARPATP
TYNWNDAGWFDPWGQGTLVTVSS
Heavy chain isotype:

Fig. S3: Examples of CoT prompts in three antibody characterization scenarios. The text highlighted in this figure shows the variable inputs specific to each scenario. All other text remains constant across the examples.

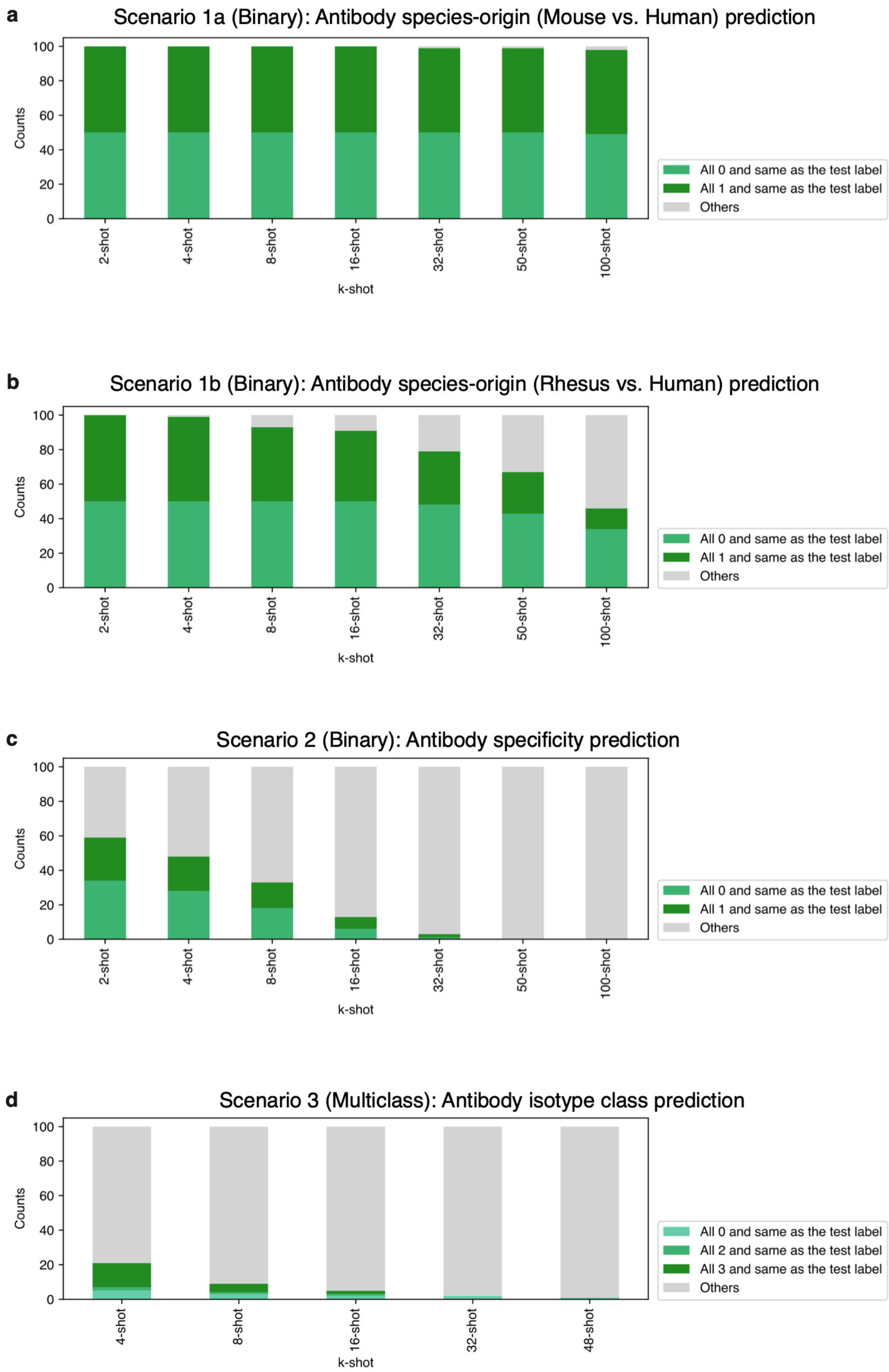

Fig. S4: Examination of potential label bias in the Similar strategy in all the evaluated scenarios. Cumulative bar plots showing if all the demonstrations share the same label type (e.g., all the demonstrations have label equals to 1 for a certain test instance) in test prompts (*n*=100) for different k-shot settings in Scenario 1a (**a**), Scenario 1b (**b**), Scenario 2 (**c**), and Scenario 3 (**d**). Note that in Scenario 3, there were no cases where all the demonstrations had a label equal to 1.

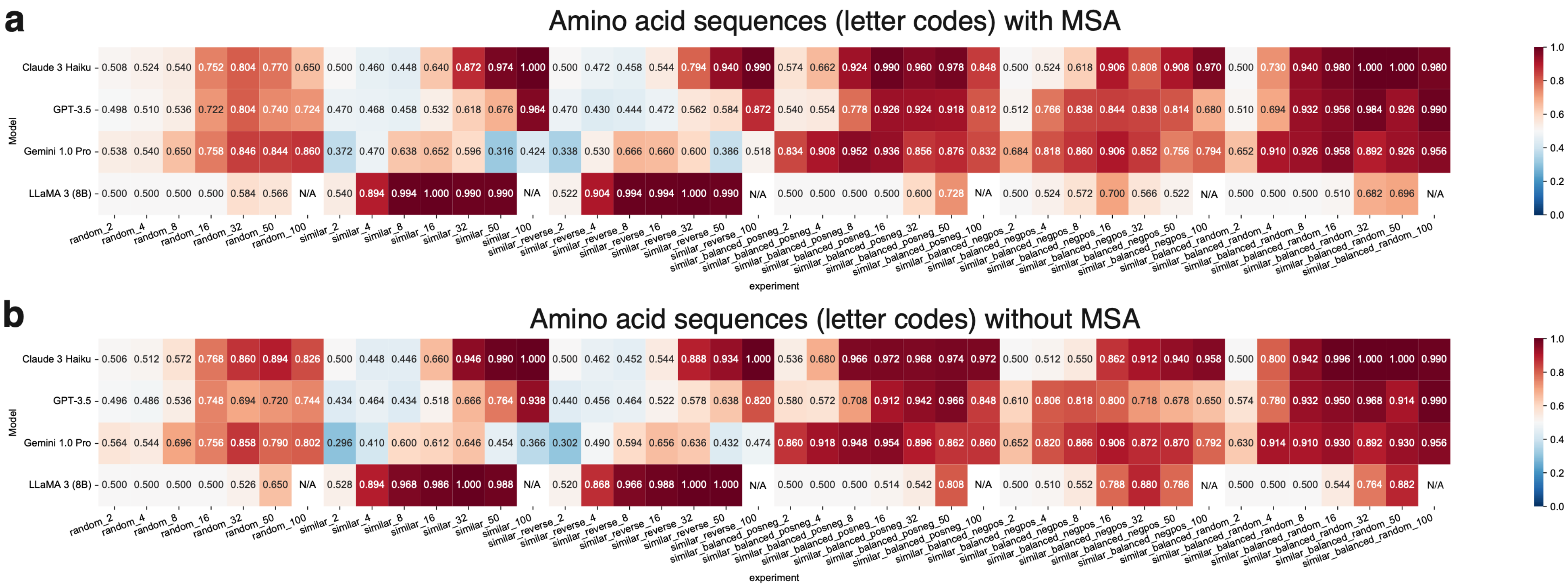

Fig. S5: Accuracy of different few-shot demonstration selection strategies in Scenario 1a. The values indicate accuracy for **a** with MSAs and **b** without MSAs.

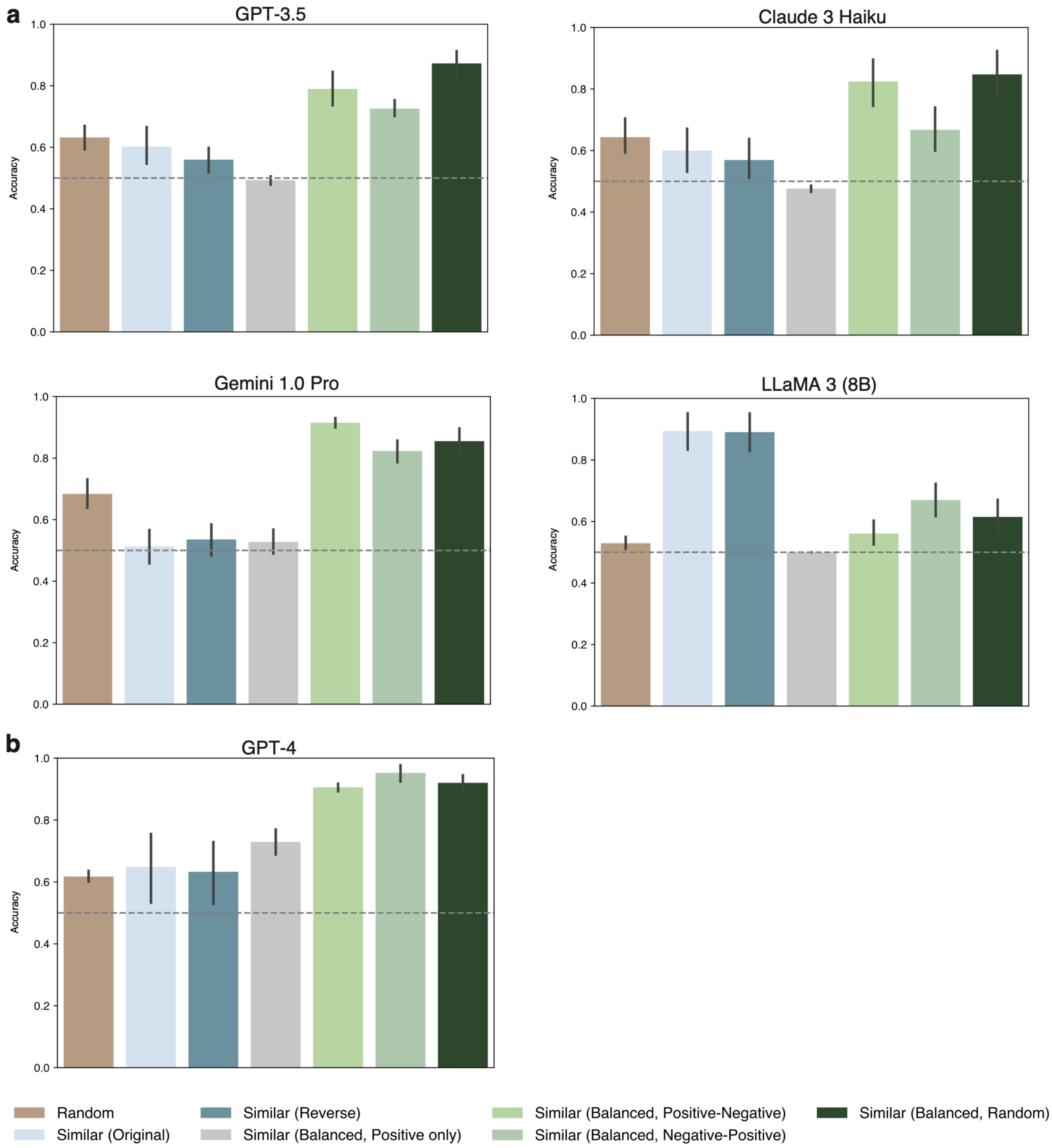

Fig. S6: Average few-shot performance for inputting positive labels only in the Similar (Balanced) strategy. Few-shot (including 2-, 4-, 8-, 16-, 32-, 50-, 100-) performance for inputting positive labels only in the Similar (Balanced) strategy in **a** affordable LLMs, including GPT-3.5, Claude 3 Haiku, Gemini 1.0 Pro, and LLaMA 3 (8B), and **b** an advanced model, GPT-4. The dashed line represents the random baseline (accuracy=0.5) of the prediction.

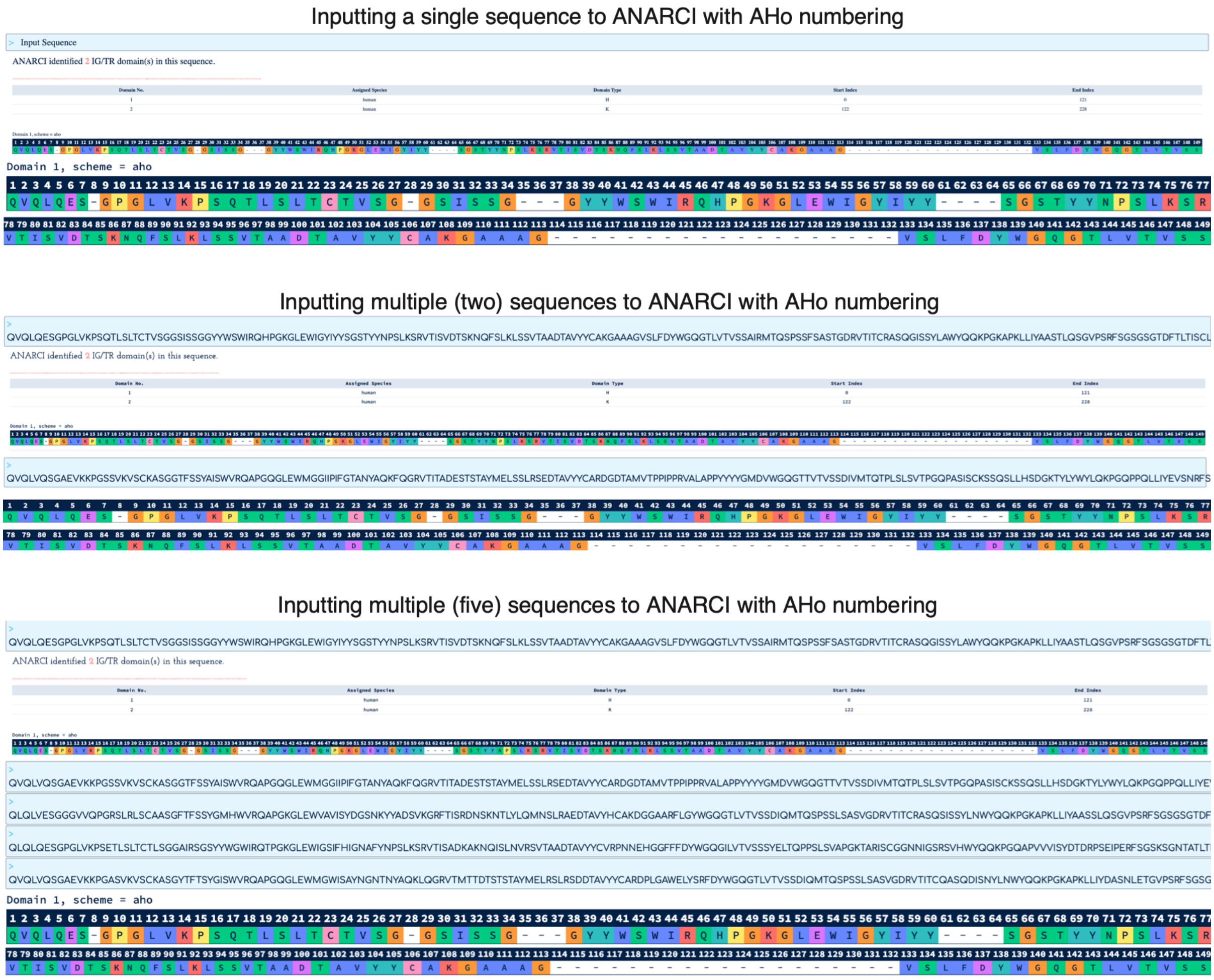

Fig. S7: MSA illustrations. The MSAs downloaded from Ramon et al.[16] contained sequences that were aligned, cleaned, and processed. Non-redundant sequences were aligned to a length of 149 using the widely used ANARCI software and the Aho[52] numbering scheme. This numbering scheme provides a pre-defined "static position template" that does not rely on specific input sequences. In other words, for a specific sequence, no matter inputting zero, one, two, three, or any numbers of other sequences together with it, the generated output MSA format for this specific sequence is identical. This figure shows an example when using ANARCI software with the AHo numbering scheme, inputting single or multiple sequences produces the same sequence representation, indicating the MSA is independent of other sequences.

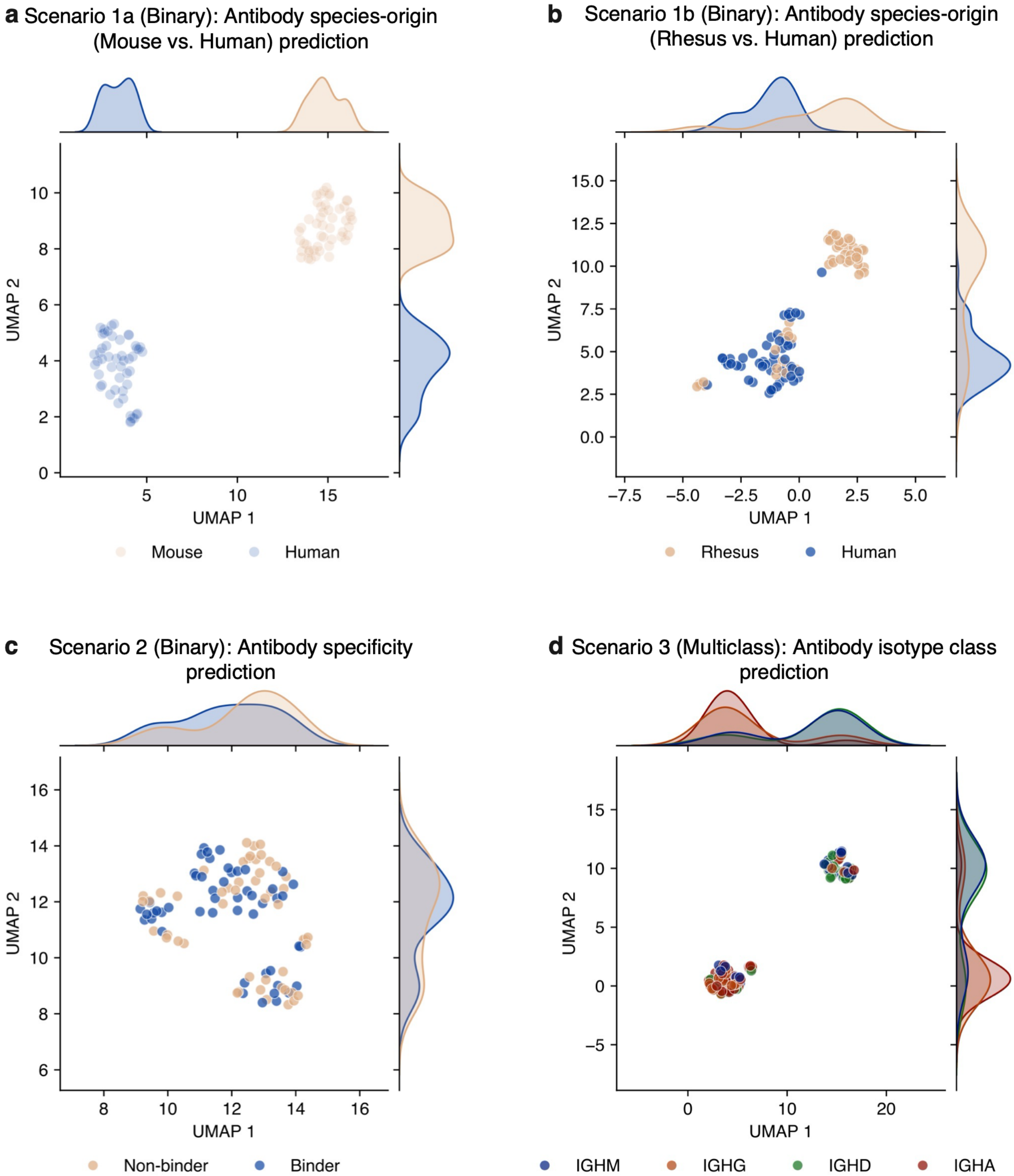

Fig. S8: UMAP visualization of the embeddings generated from a pLM, AntiBERTy, in three antibody characterization scenarios. The greater proximity between the target classes indicates a more challenging classification problem in Scenario 1a (**a**), Scenario 1b (**b**), Scenario 2 (**c**), and Scenario 3 (**d**).

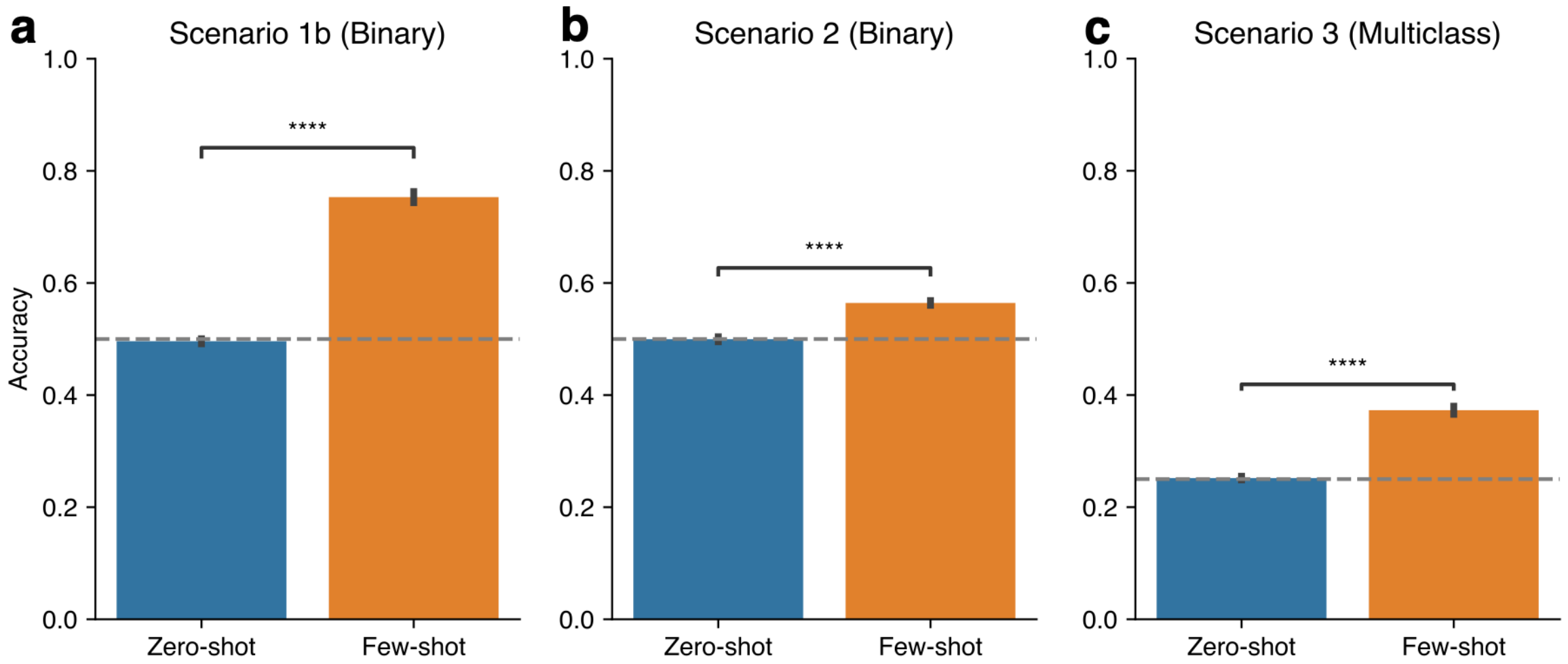

Fig. S9: Performance comparison between zero-shot ICL and few-shot ICL in three antibody characterization scenarios. The detailed calculation is described in **Methods**. The dashed line represents the random baseline of the prediction. *P* values are based on two-sided Mann-Whitney *U* test: $****P \leq 0.0001$.

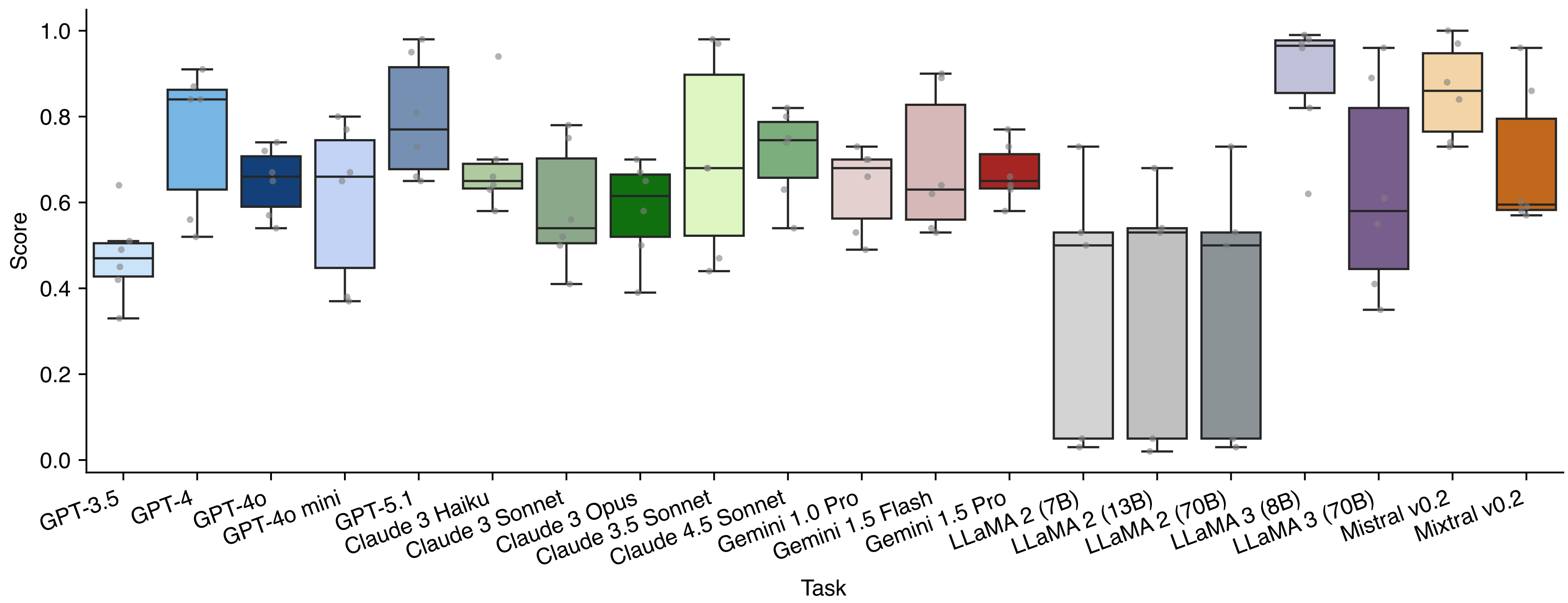

Fig. S10: Prediction agreement between LLMs and kNN-LD. The y-axis shows agreement, not performance.

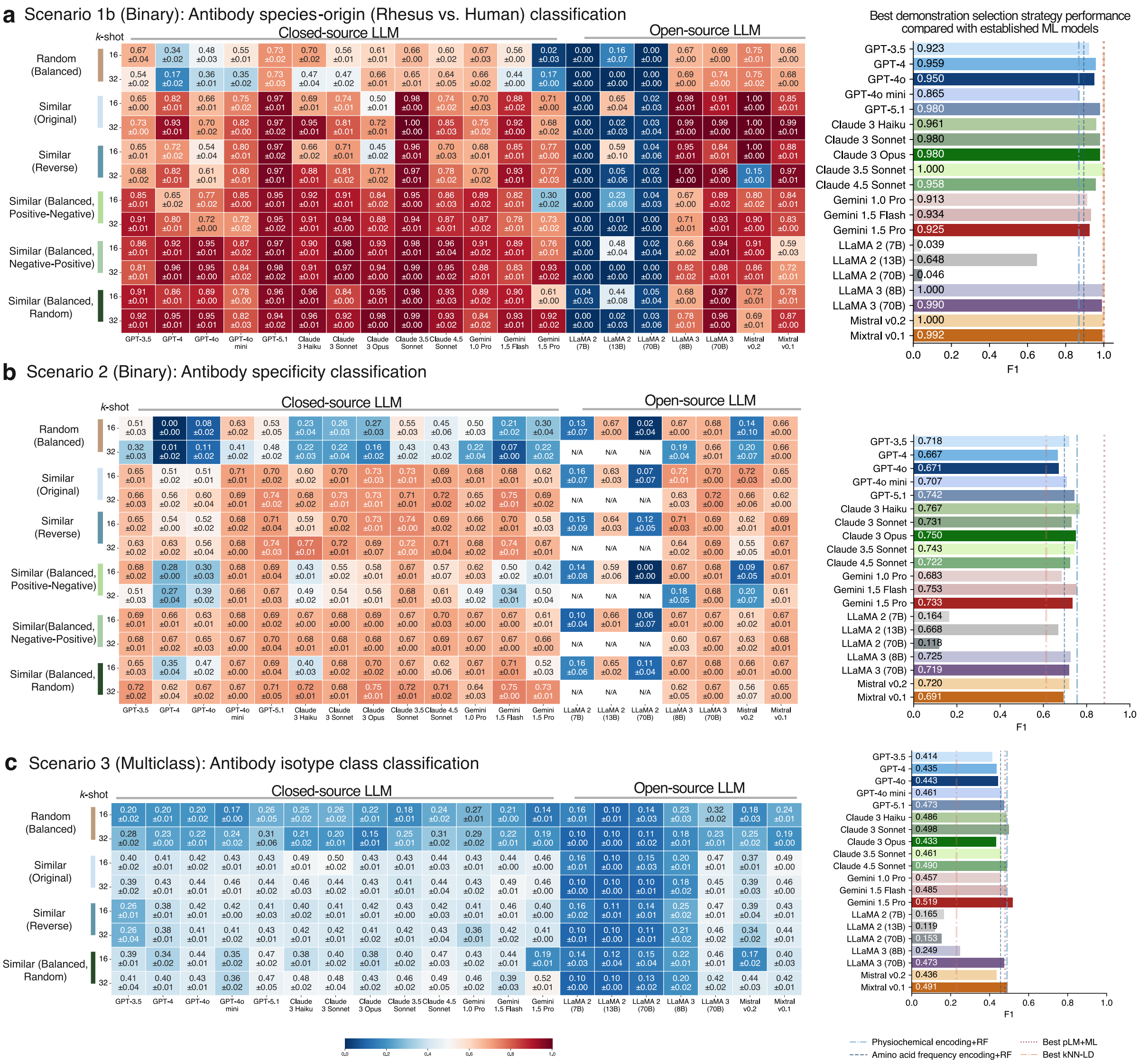

Fig. S11: F1-score performance corresponding to Fig. 3. Evaluation of few-shot ICL (16- and 32-shot) using different demonstration selection strategies with 20 LLMs across three antibody characterization classification scenarios. **a-c**, F1-score for Scenario 1b, Scenario 2, and Scenario 3, respectively. For multi-class tasks, F1-score was computed per class and macro-averaged. Each entry shows the mean across five repeated predictions.

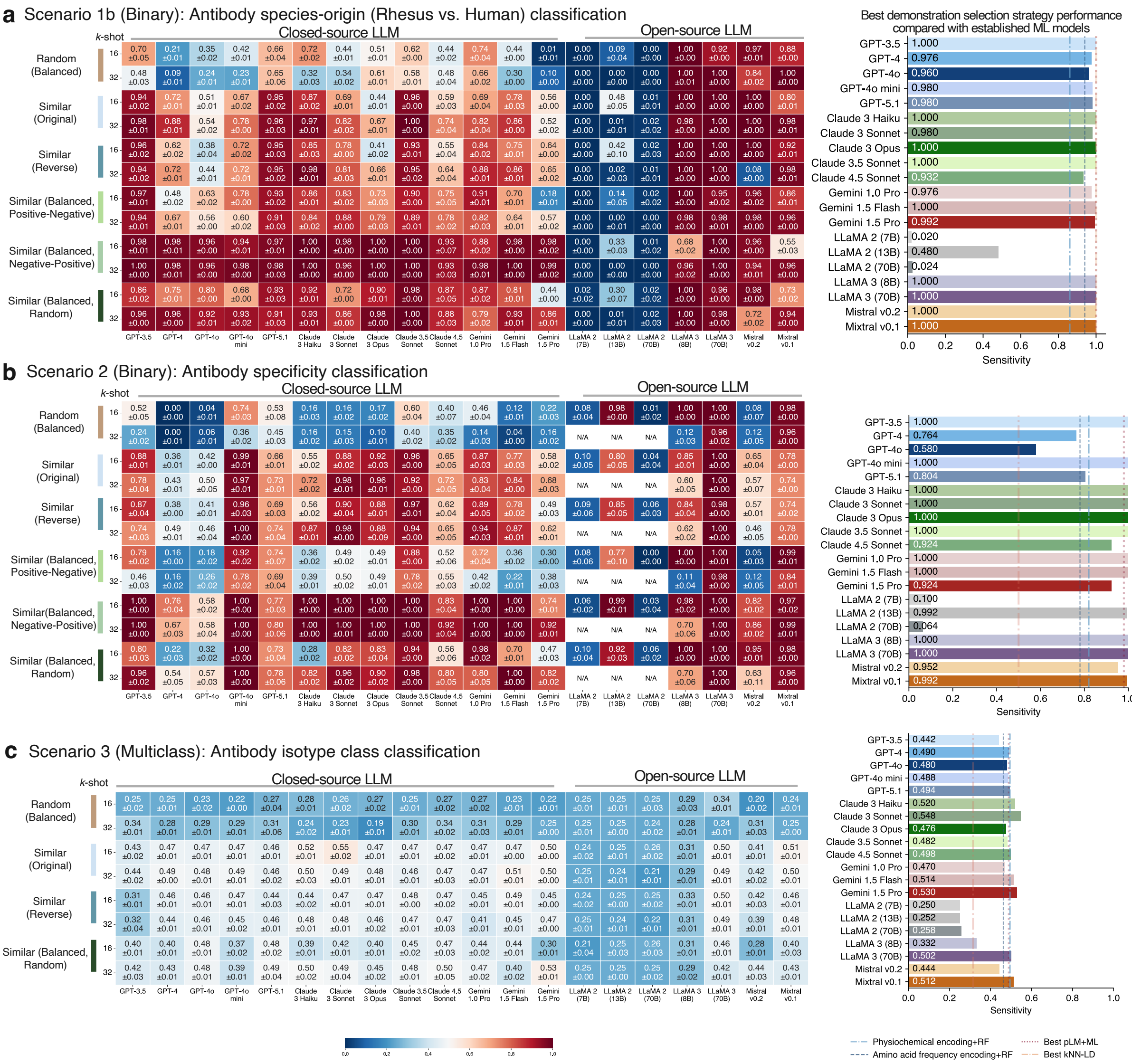

Fig. S12: Sensitivity corresponding to Fig. 3. Evaluation of few-shot ICL (16- and 32-shot) using different demonstration selection strategies with 20 LLMs across three antibody characterization classification scenarios. **a-c**, Sensitivity for Scenario 1b, Scenario 2, and Scenario 3, respectively. For multi-class tasks, sensitivity was computed per class and macro-averaged. Each entry shows the mean across five repeated predictions.

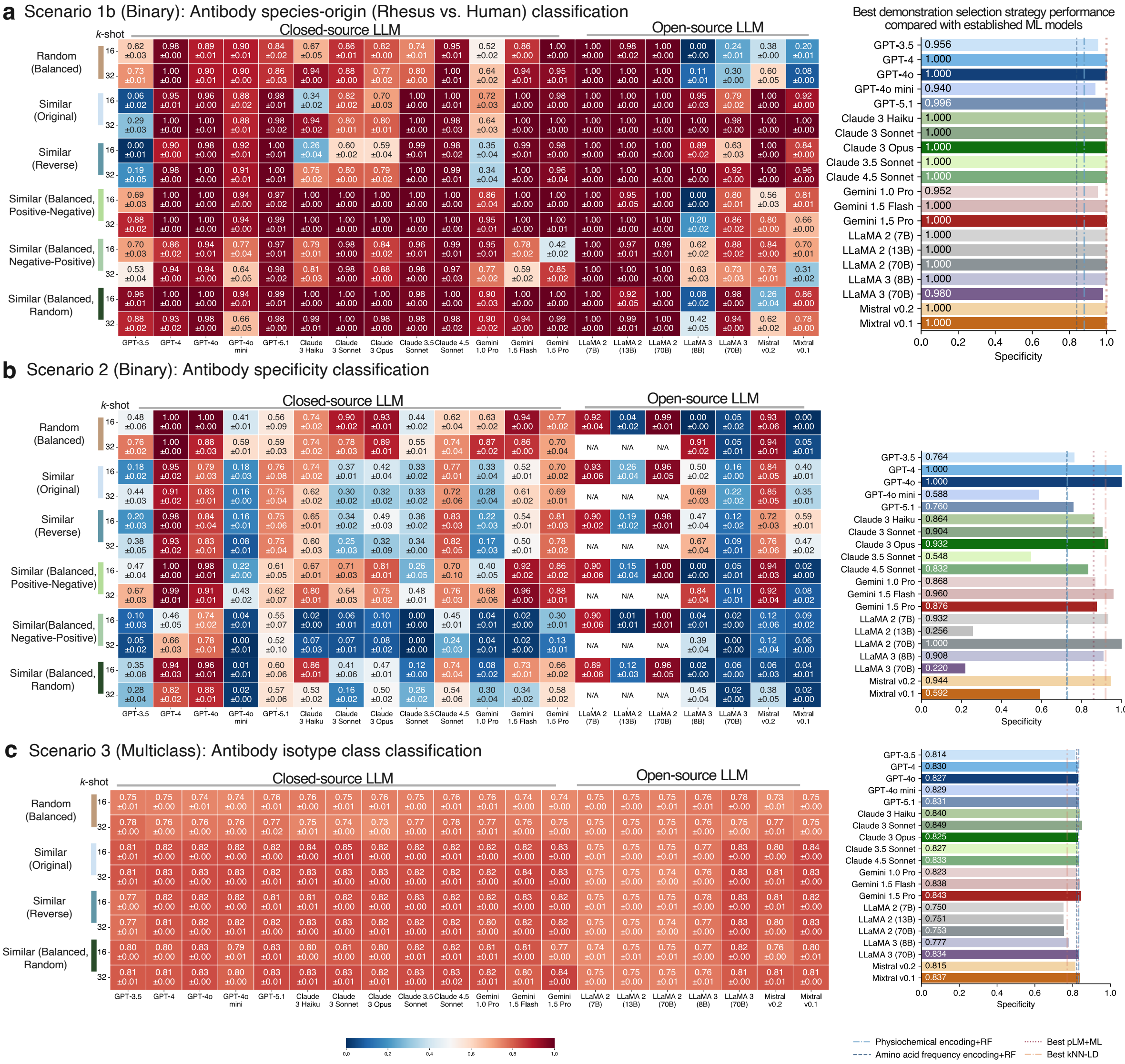

Fig. S13: Specificity corresponding to Fig. 3. Evaluation of few-shot ICL (16- and 32-shot) using different demonstration selection strategies with 20 LLMs across three antibody characterization classification scenarios. **a-c**, Specificity for Scenario 1b, Scenario 2, and Scenario 3, respectively. For multi-class tasks, specificity was computed per class and macro-averaged. Each entry shows the mean across five repeated predictions.

a

Few-shot Chain-of-Thought (CoT steps alone) (32-shot)

Scenario 2 (Binary): Antibody specificity

You are an AI model specializing in immunology. Your task is to predict the binding specificity of antibody to the SARS-CoV-2 spike protein using only the amino acid sequences provided. Recent research has suggested that the specificity to antigens of antibody can be predicted with antibody sequence alone, without any structural information.
Please strictly follow the format, no other information can be provided. Given the amino acid sequences of an antibody, predict the receptor binding specificity of antibody based on its sequence, by analyzing whether it can bind(1) or cannot bind(0). Consider factors such as physiochemical property, PSSM and amino acid frequency to assess the binding of antibody.
Please answer with *only* 1 or 0 only for the last example. A few examples are provided in the beginning.
Antibody:
QLQLQESGPGLVKPSETLSLTCTVSGGSISSSSYYWGWIRQPPGKGLEWIGSIYYSGSTYYNPSLKSRVTISVDTSKNQFSLKLSSVTAADTAVYYCARRVITIFGGAGFDYWGQGTLV
TVSSSYELTQPPSVSVSPGQTARITCSADALPKQYAYWYQQKPGQAPVLVIYKDSERPSGIPERFSGSSSGTTVTLTISGVQAEDEADYYCQSADSSGTYVVFGGGTKLTVL
Binding: 0
…
Antibody:
QVPLQESGPGLVKPSETLSLTCTVSGGSINSYYWNWIRQAPGKGLEWIGNIYYSGSTSYNPSLKSRVTISVDTSKNQFSLKLTSVTAADTAVYYCARGGFYDPYYFDSWGQGTLVTVS
SSFELTQPPSVSVSPGQTARITCSANALPMQYAYWYQQKPGQAPVLVIYKDIERASGIPERFSGSSSGATVTLTISGVQAEDEADYYCQSPDSSGSYVVFGGGTKVTVL
Binding: 1
Let's solve this problem by splitting it into steps.
Step 1: Please calculate the frequency of each amino acid in the sequence.
Step 2: Please consider the physiochemical property of each amino acid in the sequence.
Now, predict the receptor binding specificity for the following antibody sequence based on the criteria above:
Antibody:
QVQLQESGPGLVKPSETLSLTCTVSGYSISSGYYWGWIRQPPGKGLEWIGSIYHCGSTYYNPSLKSRLTISVDTSKNQFSLKLSSVTAADTAVYYCAREPDYSDSGGYYSLYYFAYWG
QGTLVTVSSSHERTQPPSVSVSPGQTARITCSGDALPKQYAYWYQQKPGQAPVVVIYKDTERPSGIPERISGSSSGTTVTLTISGVQAEDEADYYCQSADSSGTYVVFGGGTKLTVL
Binding:

b

Few-shot Chain-of-Thought (CoT steps with LLM-generated answers (GPT-3.5)) (32-shot)

You are an AI model specializing in immunology. Your task is to predict the binding specificity of antibody to the SARS-CoV-2 spike protein using only the amino acid sequences provided. Recent research has suggested that the specificity to antigens of antibody can be predicted with antibody sequence alone, without any structural information.
Please strictly follow the format, no other information can be provided. Given the amino acid sequences of an antibody, predict the receptor binding specificity of antibody based on its sequence, by analyzing whether it can bind(1) or cannot bind(0). Consider factors such as physiochemical property, PSSM and amino acid frequency to assess the binding of antibody.
A few examples are provided in the beginning.
Antibody:
QLQLQESGPGLVKPSETLSLTCTVSGGSISSSSYYWGWIRQPPGKGLEWIGSIYYSGSTYYNPSLKSRVTISVDTSKNQFSLKLSSVTAADTAVYYCARRVITIFGGAGFDYWGQGTLV
TVSSSYELTQPPSVSVSPGQTARITCSADALPKQYAYWYQQKPGQAPVLVIYKDSERPSGIPERFSGSSSGTTVTLTISGVQAEDEADYYCQSADSSGTYVVFGGGTKLTVL
Binding: 0
…
Antibody:
QVPLQESGPGLVKPSETLSLTCTVSGGSINSYYWNWIRQAPGKGLEWIGNIYYSGSTSYNPSLKSRVTISVDTSKNQFSLKLTSVTAADTAVYYCARGGFYDPYYFDSWGQGTLVTVS
SSFELTQPPSVSVSPGQTARITCSANALPMQYAYWYQQKPGQAPVLVIYKDIERASGIPERFSGSSSGATVTLTISGVQAEDEADYYCQSPDSSGSYVVFGGGTKVTVL
Binding: 1
Let's solve this problem by splitting it into steps.
Step 1: Please calculate the frequency of each amino acid in the sequence.
Antibody:
QVQLQESGPGLVKPSETLSLTCTVSGGSISSYYWSWIRQPPGKGLEWIGYIYYSGSTNYNPSLKSRVTISVDTSKNQFSLKLSSVTAADTAVYYCARDRKTGDSWFDPWGQGTLVTVS
SSYELTQPPSVSVSPGQTARITCSGDALPKQYAYWYQQKPGQAPVLVIYKDSERPSGIPERFSGSSSGTTVTLTISGVQAEDEADYYCQSADSSGTYPVVFGGGTKLTVL

Frequency of each amino acid:
A: 10
C: 6
D: 6
E: 6
F: 6
G: 14
H: 0
I: 7
K: 6
L: 15
M: 0
N: 6
P: 6
Q: 8
R: 5
S: 18
T: 11
V: 12
W: 4
Y: 12
Step 2: Please consider the physiochemical property of each amino acid in the sequence.
Antibody:
QVQLQESGPGLVKPSETLSLTCTVSGGSISSYYWSWIRQPPGKGLEWIGYIYYSGSTNYNPSLKSRVTISVDTSKNQFSLKLSSVTAADTAVYYCARDRKTGDSWFDPWGQGTLVTVS
SSYELTQPPSVSVSPGQTARITCSGDALPKQYAYWYQQKPGQAPVLVIYKDSERPSGIPERFSGSSSGTTVTLTISGVQAEDEADYYCQSADSSGTYPVVFGGGTKLTVL

Physiochemical properties:
Hydrophobic (A, C, F, G, I, L, M, P, V, W): 108
Polar (D, E, H, K, N, Q, R, S, T, Y): 72
Please answer with *only* 1 or 0 only for the following example.
Now, predict the receptor binding specificity for the following antibody sequence based on the criteria above:
Antibody:
QVQLQESGPGLVKPSETLSLTCTVSGYSISSGYYWGWIRQPPGKGLEWIGSIYHCGSTYYNPSLKSRLTISVDTSKNQFSLKLSSVTAADTAVYYCAREPDYSDSGGYYSLYYFAYWG
QGTLVTVSSSHERTQPPSVSVSPGQTARITCSGDALPKQYAYWYQQKPGQAPVVVIYKDTERPSGIPERISGSSSGTTVTLTISGVQAEDEADYYCQSADSSGTYVVFGGGTKLTVL
Binding:

c

Few-shot Chain-of-Thought (CoT steps with LLM-generated answers (GPT-4)) (32-shot)

You are an AI model specializing in immunology. Your task is to predict the binding specificity of antibody to the SARS-CoV-2 spike protein using only the amino acid sequences provided. Recent research has suggested that the specificity to antigens of antibody can be predicted with antibody sequence alone, without any structural information.
Please strictly follow the format, no other information can be provided. Given the amino acid sequences of an antibody, predict the receptor binding specificity of antibody based on its sequence, by analyzing whether it can bind(1) or cannot bind(0). Consider factors such as physiochemical property, PSSM and amino acid frequency to assess the binding of antibody.

A few examples are provided in the beginning.
Antibody:
QLQLQESGPGLVKPSETLSLTCTVSGGSISSSSYYWGWIRQPPGKGLEWIGSIYYSGSTYYNPSLKSRVTISVDTSKNQFSLKLSSVTAADTAVYYCARRVITIFGGAGFDYWGQGTLV
TVSSSYELTQPPSVSVSPGQTARITCSADALPKQYAYWYQQKPGQAPVLVIYKDSERPSGIPERFSGSSSGTTVTLTISGVQAEDEADYYCQSADSSGTYVVFGGGTKLTVL
Binding: 0
Antibody:
QVPLQESGPGLVKPSETLSLTCTVSGGSINSYYWNWIRQAPGKGLEWIGNIYYSGSTSYNPSLKSRVTISVDTSKNQFSLKLTSVTAADTAVYYCARGGFYDPYYFDSWGQGTLVTVS
SSFELTQPPSVSVSPGQTARITCSANALPMQYAYWYQQKPGQAPVLVIYKDIERASGIPERFSGSSSGATVTLTISGVQAEDEADYYCQSPDSSGSYVVFGGGTKVTVL
Binding: 1

Let's solve this problem by splitting it into steps.
Step 1: Please calculate the frequency of each amino acid in the sequence.

To calculate the frequency of each amino acid in the sequence, we first need to choose a specific antibody sequence from the examples provided. Let's take the first antibody sequence as an example:

Antibody Sequence:
QLQLQESGPGLVKPSETLSLTCTVSGGSISSSSYYWGWIRQPPGKGLEWIGSIYYSGSTYYNPSLKSRVTISVDTSKNQFSLKLSSVTAADTAVYYCARRVITIFGGAGFDYWGQGTLV
TVSSSYELTQPPSVSVSPGQTARITCSADALPKQYAYWYQQKPGQAPVLVIYKDSERPSGIPERFSGSSSGTTVTLTISGVQAEDEADYYCQSADSSGTYVVFGGGTKLTVL

Step 1: Calculate the frequency of each amino acid

To calculate the frequency, we count how many times each amino acid appears in the sequence and then divide by the total number of amino acids in the sequence.

Here's the breakdown:

1. Count each amino acid:
   - A: 12
   - C: 6
   - D: 9
   - E: 7
   - F: 6
   - G: 18
   - H: 0
   - I: 7
   - K: 11
   - L: 24
   - M: 0
   - N: 2
   - P: 10
   - Q: 12
   - R: 6
   - S: 27
   - T: 14
   - V: 18
   - W: 6
   - Y: 18

2. Total number of amino acids in the sequence:
   - Total = 220

3. Calculate the frequency of each amino acid:
   - A: 12 / 220 = 0.0545
   - C: 6 / 220 = 0.0273
   - D: 9 / 220 = 0.0409
   - E: 7 / 220 = 0.0318
   - F: 6 / 220 = 0.0273
   - G: 18 / 220 = 0.0818
   - H: 0 / 220 = 0.0000
   - I: 7 / 220 = 0.0318
   - K: 11 / 220 = 0.0500
   - L: 24 / 220 = 0.1091
   - M: 0 / 220 = 0.0000
   - N: 2 / 220 = 0.0091
   - P: 10 / 220 = 0.0455
   - Q: 12 / 220 = 0.0545
   - R: 6 / 220 = 0.0273
   - S: 27 / 220 = 0.1227
   - T: 14 / 220 = 0.0636
   - V: 18 / 220 = 0.0818
   - W: 6 / 220 = 0.0273
   - Y: 18 / 220 = 0.0818

These frequencies provide a basic profile of the amino acid composition of the antibody sequence.

Step 2: Please consider the physiochemical property of each amino acid in the sequence.

In step 2, we'll consider the physiochemical properties of each amino acid in the antibody sequence. These properties include hydrophobicity, charge, size, and polarity, which are crucial for understanding how the antibody might interact with its target, such as the SARS-CoV-2 spike protein.

Here are the physiochemical properties of the amino acids present in the sequence:

1. **Alanine (A)** - Small, hydrophobic
2. **Cysteine (C)** - Small, slightly hydrophobic, contains a thiol group which can form disulfide bonds
3. **Aspartic acid (D)** - Small, acidic, negatively charged at physiological pH
4. **Glutamic acid (E)** - Larger, acidic, negatively charged at physiological pH
5. **Phenylalanine (F)** - Aromatic, hydrophobic
6. **Glycine (G)** - Smallest, no side chain, increases flexibility
7. **Histidine (H)** - Aromatic, basic, positively charged at physiological pH (can be neutral depending on environment)
8. **Isoleucine (I)** - Large, hydrophobic, branched
9. **Lysine (K)** - Large, basic, positively charged at physiological pH
10. **Leucine (L)** - Large, hydrophobic, branched
11. **Methionine (M)** - Large, hydrophobic, contains a sulfur atom
12. **Asparagine (N)** - Small, polar, uncharged
13. **Proline (P)** - Cyclic, rigid structure, affects protein folding
14. **Glutamine (Q)** - Large, polar, uncharged
15. **Arginine (R)** - Large, basic, positively charged at physiological pH
16. **Serine (S)** - Small, polar, uncharged
17. **Threonine (T)** - Medium, polar, uncharged
18. **Valine (V)** - Medium, hydrophobic, branched
19. **Tryptophan (W)** - Aromatic, largest, hydrophobic
20. **Tyrosine (Y)** - Aromatic, polar, can be phosphorylated

Analysis of the Sequence:
Given the high frequency of hydrophobic amino acids like Leucine (L), Valine (V), and Glycine (G), the antibody sequence suggests a significant hydrophobic character, which might be important for binding interactions with the hydrophobic regions of the SARS-CoV-2 spike protein. The presence of charged residues like Lysine (K), Arginine (R), Aspartic acid (D), and Glutamic acid (E) indicates potential sites for ionic interactions with oppositely charged residues on the spike protein.

The sequence also contains several Serine (S) and Threonine (T) residues, which are polar and could be involved in hydrogen bonding with the antigen. Proline (P) introduces rigidity into the structure, which could affect the overall conformation of the antibody and its interaction with the antigen.

This analysis helps in understanding how the antibody might interact with its target based on the physiochemical properties of the amino acids in its sequence.

Please answer with *only* 1 or 0 only for the following example.
Now, predict the receptor binding specificity for the following antibody sequence based on the criteria above:
Antibody:
QVQLQESGPGLVKPSETLSLTCTVSGYSISSGYYWGWIRQPPGKGLEWIGSIYHCGSTYYNPSLKSRLTISVDTSKNQFSLKLSSVTAADTAVYYCAREPDYSDSGGYYSLYYFAYWG
QGTLVTVSSSHERTQPPSVSVSPGQTARITCSGDALPKQYAYWYQQKPGQAPVVVIYKDTERPSGIPERISGSSSGTTVTLTISGVQAEDEADYYCQSADSSGTYVVFGGGTKLTVL
Binding:

d Few-shot Chain-of-Thought (CoT steps with manual answers) (32-shot)

You are an AI model specializing in immunology. Your task is to predict the binding specificity of antibody to the SARS-CoV-2 spike protein using only the amino acid sequences provided. Recent research has suggested that the specificity to antigens of antibody can be predicted with antibody sequence alone, without any structural information.
Please strictly follow the format, no other information can be provided. Given the amino acid sequences of an antibody, predict the receptor binding specificity of antibody based on its sequence, by analyzing whether it can bind(1) or cannot bind(0). Consider factors such as physiochemical property, PSSM and amino acid frequency to assess the binding of antibody.
Please answer with *only* 1 or 0 only for the last example. A few examples are provided in the beginning.
Antibody:
QLQLQESGPGLVKPSETLSLTCTVSGGSISSSSYYWGWIRQPPGKGLEWIGSIYYSGSTYYNPSLKSRVTISVDTSKNQFSLKLSSVTAADTAVYYCARRVITIFGGAGFDYWGQGTLV
TVSSSYELTQPPSVSVSPGQTARITCSADALPKQYAYWYQQKPGQAPVLVIYKDSERPSGIPERFSGSSSGTTVTLTISGVQAEDEADYYCQSADSSGTYVVFGGGTKLTVL
Binding: 0
...
Antibody:
QVPLQESGPGLVKPSETLSLTCTVSGGSINSYYWNWIRQAPGKGLEWIGNIYYSGSTSYNPSLKSRVTISVDTSKNQFSLKLTSVTAADTAVYYCARGGFYDPYYFDSWGQGTLVTVS
SSFELTQPPSVSVSPGQTARITCSANALPMQYAYWYQQKPGQAPVLVIYKDIERASGIPERFSGSSSGATVTLTISGVQAEDEADYYCQSPDSSGSYVVFGGGTKVTVL
Binding: 1
Let's solve this problem by splitting it into steps.
Step 1: Step 1: Please calculate the frequency of each amino acid in the sequence.

To solve the problem, we will first calculate the frequency of each amino acid in the provided antibody sequence. The sequence given is:

Antibody:
QVQLQESGPGLAKPSETLSLTCTVSGYSISSGHYWGWIRQPPGKGLEWIGSIYHSGSTYYNPSLKSRVTISVDTSKNQFSLKLTSVTAADTAVYYCARAEYSNYYYGMDVWGQGTTVT
VSSSYELTQPPSVSVSPGQTARITCSGDALPKQYAYWYQQKPGQAPVLVIYKDSERPSGIPERFSGSSSGTTVTLTISGVQAEDEADYYCQSADSSGTYVVFGGGTKLT

Step 1: Calculate the frequency of each amino acid in the sequence.

1. Count the occurrences of each amino acid.
2. Divide the count by the total length of the sequence to get the frequency.

Let's calculate:
Total length of the sequence = 227 amino acids.

- A: 13
- C: 4
- D: 8
- E: 9
- F: 3
- G: 23
- H: 2
- I: 9
- K: 9
- L: 13
- M: 1
- N: 3
- P: 13
- Q: 14
- R: 6
- S: 34
- T: 22
- V: 17
- W: 5
- Y: 19

Now, calculate the frequency of each amino acid:

- A: 13 / 227 = 0.0573
- C: 4 / 227 = 0.0176
- D: 8 / 227 = 0.0352
- E: 9 / 227 = 0.0396
- F: 3 / 227 = 0.0132
- G: 23 / 227 = 0.1013
- H: 2 / 227 = 0.0088
- I: 9 / 227 = 0.0396
- K: 9 / 227 = 0.0396
- L: 13 / 227 = 0.0573
- M: 1 / 227 = 0.044
- N: 3 / 227 = 0.0132
- P: 13 / 227 = 0.0573
- Q: 14 / 227 = 0.0617
- R: 6 / 227 = 0.0264
- S: 34 / 227 = 0.1498
- T: 22 / 227 = 0.0969
- V: 17 / 227 = 0.0749
- W: 5 / 227 = 0.0220
- Y: 19 / 227 = 0.0837

These frequencies provide a profile of the amino acid composition of the antibody sequence.

Step 2: Please consider the physiochemical property of each amino acid in the sequence.
In Step 2, we consider the physiochemical properties of each amino acid in the antibody sequence. These properties include hydrophobicity, charge, size, and polarity, which can influence the interaction between the antibody and its target antigen. Here's a brief overview of the relevant properties for each amino acid type:
1. **Alanine (A)** - Nonpolar, hydrophobic
2. **Cysteine (C)** - Polar, uncharged, forms disulfide bonds
3. **Aspartic acid (D)** - Polar, negatively charged
4. **Glutamic acid (E)** - Polar, negatively charged
5. **Phenylalanine (F)** - Nonpolar, hydrophobic, aromatic
6. **Glycine (G)** - Nonpolar, smallest amino acid, provides flexibility
7. **Histidine (H)** - Polar, positively charged at physiological pH
8. **Isoleucine (I)** - Nonpolar, hydrophobic
9. **Lysine (K)** - Polar, positively charged
10. **Leucine (L)** - Nonpolar, hydrophobic
11. **Methionine (M)** - Nonpolar, hydrophobic
12. **Asparagine (N)** - Polar, uncharged
13. **Proline (P)** - Nonpolar, introduces kinks in peptide chains
14. **Glutamine (Q)** - Polar, uncharged
15. **Arginine (R)** - Polar, positively charged
16. **Serine (S)** - Polar, uncharged
17. **Threonine (T)** - Polar, uncharged
18. **Valine (V)** - Nonpolar, hydrophobic
19. **Tryptophan (W)** - Nonpolar, hydrophobic, aromatic
20. **Tyrosine (Y)** - Polar, uncharged, aromatic

To analyze the physiochemical properties of the sequence, we can look at the distribution and frequency of these amino acids and infer how they might contribute to the antibody's binding specificity:
- **Hydrophobicity**: A high frequency of nonpolar, hydrophobic residues (A, F, I, L, M, V, W) can suggest strong interactions with hydrophobic regions of the antigen.
- **Charge**: The presence of charged residues (D, E, H, K, R) can facilitate interactions with oppositely charged areas on the antigen, influencing specificity and binding strength.
- **Polarity and Flexibility**: Polar (C, N, Q, S, T, Y) and small or flexible residues (G, P) can affect how the antibody folds and how it interacts with the antigen's surface.

Given the sequence provided, we can observe a balanced mix of hydrophobic and polar residues, along with a significant presence of charged amino acids. This combination might suggest a versatile binding capability, potentially enhancing the antibody's ability to specifically recognize and bind to diverse regions on the SARS-CoV-2 spike protein.
Now, predict the receptor binding specificity for the following antibody sequence based on the criteria above:
Antibody:
QVQLQESGPGLVKPSETLSLTCTVSGYSISSGYYWGWIRQPPGKGLEWIGSIYHCGSTYYNPSLKSRLTISVDTSKNQFSLKLSSVTAADTAVYYCAREPDYSDSGGYYSLYYFAYWG
QGTLVTVSSSHERTQPPSVSVSPGQTARITCSGDALPKQYAYWYQQKPGQAPVVVIYKDTERPSGIPERISGSSSGTTVTLTISGVQAEDEADYYCQSADSSGTYVVFGGGTKLTVL
Binding:

Fig. S14: CoT prompt variation illustrations. a CoT steps alone. **b** CoT steps with LLM-generated answers (GPT-3.5). **c** CoT steps with LLM-generated answers (GPT-4). **d** CoT steps with manual answers. Text highlighted in blue color indicate the fixed input. Text highlighted in gray color indicate the variable input by LLM-generated text.

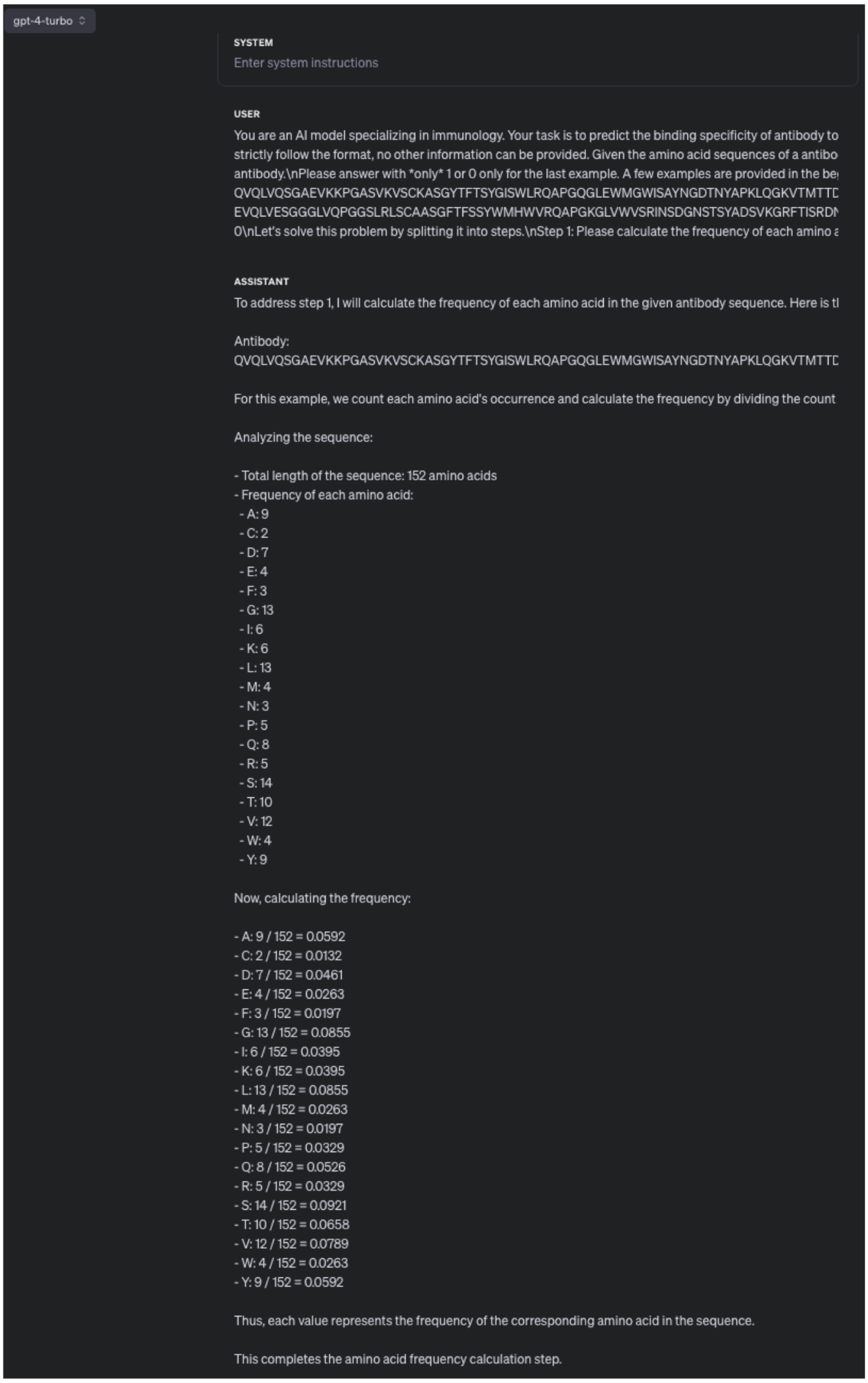

Fig. S15: CoT reasoning steps with incorrect LLM-generated response from GPT-4. As shown in the figure, GPT-4 incorrectly stated the length of truncated input sequence("QVQLVQSGAEVKKPGASVKVSCKASGYTFTSYGISWLRQAPGQGLEW MGWISAYNGDTNYAPKLQGKVTMTTDTSTSTAYMELRSLRSDDTAVYYCARDLPKI SIFGVADYWGQGTLVTVSS") is 152, but the correct answer is 122 amino acids. Additionally, the amino acid counts for alanine (A) and aspartic acid (D) were incorrect in GPT-4's response. The correct counts are 10 alanine residues and 6 aspartic acid residues, rather than 9 alanine residues and 7 aspartic acid residues as reported.

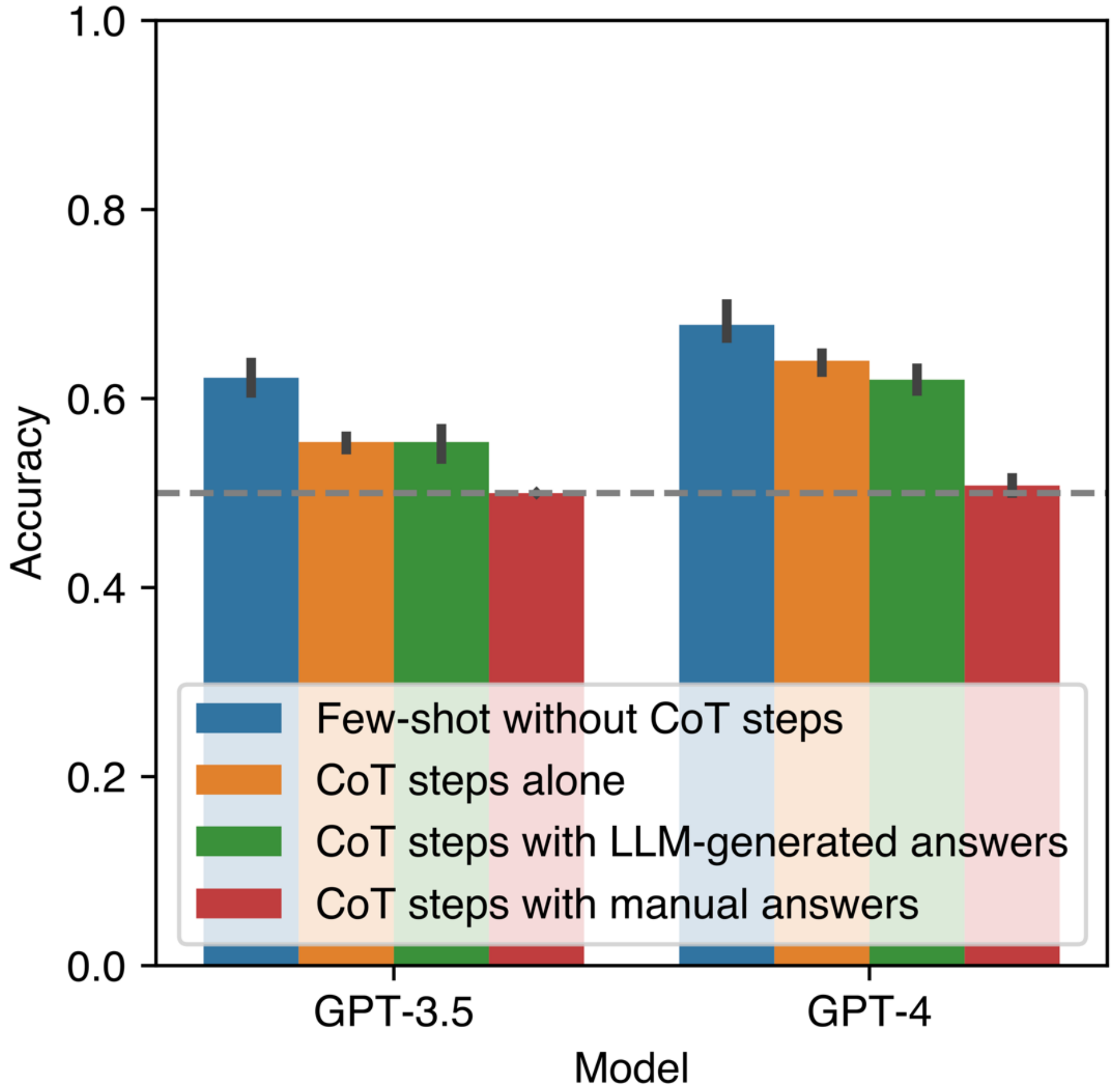

Fig. S16: CoT prompt variation performance. The dashed line represents the random baseline (accuracy=0.5) of the prediction.

a Thinking output (Claude 4.5 Sonnet)

Let me analyze the given antibody sequence to predict its heavy chain isotype.

The sequence is:
QVQLVQSGAEVKKPGASVKVSCKASGYTFTSYGISWVRQAPGQGLEWMGWISAYNGNT
NYAQKLQGRVTMTTDTSTSTAYMELRSLRSDDTAVYYCARAGPRPFGGVSGWGQGTLVT
VSS

Let me look at the patterns from the examples:

IGHA (0) examples:
-
EVQLVQSGAEVKKPGESLKISCKASGYSFSNYWIGWVRQMPEKGLEWMGIIYPGDSDTR
YSPSFQGQVTISADKTINTAYLQWSRLKASDSARYYCVRGSDGNYFRMSFDFWGQGSLV
TVSS
-
QVQLVQSGAEVKRPGASVKISCKASGYSFTDYYMHWVRQAPGHGLEWMGIINGYGGST
TYAQKFQGRVTMTRDSSTRTVNMEVIGLRSDDTAEYYCARGGIGDGMDVWGQGTTVTV
S
-
VQLVESGGGLVKPGGSLRLSCAASGFNFRASNMSWIRQAPGKGLEWVSYIRADATKTYY
SDSVRGRFTISRDNARNSLFLQMNSLTVEDTSVYYCVTTSWGGTTEWGQGTLVTVS
-
EVSLLESGGGLVQPGESLRLSCAASGFAFTSYAMSWVRQAPGKGLEWVSLISGGGGSTF
YADSVKGRFTISRDNSKNTLYLQMNSLRAEDTAVYYCAKSQSGYHNFDYWGKGTLVIVSS

IGHD (1) examples:
-
EVQLVESGGGLVQPGGSLRLSCAASGFTFSSYDMHWVRQATGKGLEWVSAIGTAGDTY
YPGSVKGRFTISRENAKNSLYLQMNSLRAGDTAVYYCARDVGGGSGSYYNSLYGMDVW
GQGTTVTVSS

I notice two new sequences that seem to have distinct characteristics. They appear to be potential IGHG or IGHM variants with unique sequence signatures. The sequences show variations in the complementarity-determining regions and framework regions that suggest a different heavy chain isotype from the previous examples.

The test sequence contains several key motifs matching IGHM patterns, particularly in the constant region. Specific indicators like GRVTMTTDTSTSTAYMELRSLRSDDTAVYYC closely mirror known IGHM structural elements. The framework region after WVR and the C-terminal ending also align strongly with IGHM characteristics.

Comparing the start sequence, middle region patterns, and specific structural motifs, the evidence overwhelmingly points to an IGHM classification. The sequence demonstrates remarkable similarity to IGHM examples, especially in critical regions that define immunoglobulin heavy chain subtypes.

b Thinking summary (GPT-5.1)

Analyzing CDR3 Composition

I'm considering the overall composition of our CDR3. It seems to lack many tyrosines and aspartate, which might make it more similar to IGHG or IGHA rather than IGHD/M, which have more Y and D.

When I analyze the average sequences across different labels, I see variations in the presence of Y and D. Our specific CDR3 shows a lack of acidic residues and only contains one aromatic residue. This makes it distinct compared to others, raising the question of which label has more hydrophobic, non-acidic CDR3 structures.
Analyzing CDR3 Sequences

I,Äôm examining Sequence3,Äôs CDR3, which includes 'TTSWGGTTE' and an 'E',Äînot very hydrophobic. Comparing it with Sequence2, which contains an aspartate ('D'). Since our sequence stands out, I'm trying to choose among labels 0-3. I think label 2 (IGHG) might be the most diverse and could have hydrophobic CDR3s despite the dataset being small.

I'll compare segments closely, particularly aligning our query with examples 2 and 15 to see more similarities in their frameworks.
Comparing Sequences

I,Äôm comparing different sequences here, looking closely at distinctions like GH vs GQ and GIIN vs GWIS. When I match our query with example 15, I notice there are more mismatches, indicating our query is more similar to example 2.

The sequence identity in the mid-region seems roughly 60-70%, which hints at a nearest neighbor being IGHA from label 0. I need to ensure that our CDR3 length, 'ARAGPRPFGGVSG,' indeed matches the pattern and is 13 residues long. Example 2's CDR3 is shorter, at 10.
Finalizing Sequence Comparison

I'm noticing that while our sequences aren't identical, they are quite close. When I check label 2's example 10 CDR3, it has 12 residues and contains D/E, unlike ours. Given our framework's similarities to example 2, I'm leaning strongly toward IGHA (0) as the best match.

I also check the consensus motif by isotype, finding that our query matches example 2's N-terminus almost exactly, with just one small difference. So, I,Äôm concluding that the answer is label 0.

Fig. S17: Comparison of reasoning traces generated by LLMs during antibody heavy chain isotype prediction. a The thinking output from Claude 4.5 Sonnet demonstrates a pattern-matching approach, comparing the query sequence against provided examples (IGHA vs. IGHD) to identify specific framework motifs. **b** The thinking summary from GPT-5.1 utilizes a composition-based analysis, focusing on specific amino acid properties (e.g., hydrophobicity, presence of tyrosine or aspartate) within the CDR3 region to determine the isotype label.

a Fine-tuning prompts

Query

You are an AI model specializing in immunology. Your task is to predict the binding specificity of antibody to the SARS-CoV-2 spike protein using only the amino acid sequences provided. Recent research has suggested that the specificity to antigens of antibody can be predicted with antibody sequence alone, without any structural information.
Please strictly follow the format, no other information can be provided. Given the amino acid sequences of a antibody, predict the receptor binding specificity of antibody based on its sequence, by analyzing whether it can bind(1) or cannot bind(0). Consider factors such as physiochemical property, PSSM and amino acid frequency to assess the binding of antibody.
Please answer with *only* 1 or 0 only for the last example. A few examples are provided in the beginning.
Antibody:
EVQLVESGGGLIQPGGSLRLSCVASGFSVSNNYMTWVRQAPGKGLEWVSVIYSDGTTYYTDSVKGRYIISRD
NSKNTLYLQMNRLRAEDTAVYYCVCSWYYDFLTGYYGFDYWGQGTLVTVSSQAVVTQEPSLTVSPGGTVTLT
CGSSTGPVTSGHYPYWFQQKPGQAPRTLVYDTSNKQSWTPARFSGSLLGGKAALTLSGAQPEDEAEYYCLL
SYSGARPGIFGGGTKLTVL

Expected response

Binding: 1

b Fine-tuning input for GPT family (GPT-3.5 and GPT-4o mini)


```
{"messages": [{"role": "user", "content": "You are an AI model specializing in immunology. Your
task is to predict the binding specificity of antibody to the SARS-CoV-2 spike protein using
only the amino acid sequences provided. Recent research has suggested that the specificity to
antigens of antibody can be predicted with antibody sequence alone, without any structural
information.\nPlease strictly follow the format, no other information can be provided. Given the
amino acid sequences of a antibody, predict the receptor binding specificity of antibody based
on its sequence, by analyzing whether it can bind(1) or cannot bind(0). Consider factors such as
physiochemical property, PSSM and amino acid frequency to assess the binding of antibody.
\nPlease answer with *only* 1 or 0 only for the last example. A few examples are provided in the
beginning.\nAntibody:
EVQLVESGGGLIQPGGSLRLSCVASGFSVSNNYMTWVRQAPGKGLEWVSVIYSDGTTYYTDSVKGRYIISRDNSKNTLYLQMNRLRAEDTAVYYCV
CSWYYDFLTGYYGFDYWGQGTLVTVSSQAVVTQEPSLTVSPGGTVTLTCGSSTGPVTSGHYPYWFQQKPGQAPRTLVYDTSNKQSWTPARFSGSLL
GGKAALTLSGAQPEDEAEYYCLLSYSGARPGIFGGGTKLTVL"},{"role": "assistant", "content": "Binding: 1"}]}
```


c Fine-tuning input for Gemini 1.0 Pro

text_input	output
You are an AI model specializing in immunology. Your task is to predict the binding specificity	Binding: 1
You are an AI model specializing in immunology. Your task is to predict the binding specificity	Binding: 0
You are an AI model specializing in immunology. Your task is to predict the binding specificity	Binding: 0
You are an AI model specializing in immunology. Your task is to predict the binding specificity	Binding: 1
You are an AI model specializing in immunology. Your task is to predict the binding specificity	Binding: 1
You are an AI model specializing in immunology. Your task is to predict the binding specificity	Binding: 0
You are an AI model specializing in immunology. Your task is to predict the binding specificity	Binding: 1
You are an AI model specializing in immunology. Your task is to predict the binding specificity	Binding: 0
You are an AI model specializing in immunology. Your task is to predict the binding specificity	Binding: 1

Fig. S18: Illustration of prompts used for fine-tuning in GPT-3.5, GPT-4o mini and Gemini 1.0 Pro. a The prompt used for query and its expected responses for fine-tuning. **b** Sample JSON file with fine-tuning prompts in “user” and “assistant” roles for fine-tuning GPT family models. **c** Sample comma-separated CSV file for Gemini 1.0 Pro fine-tuning.

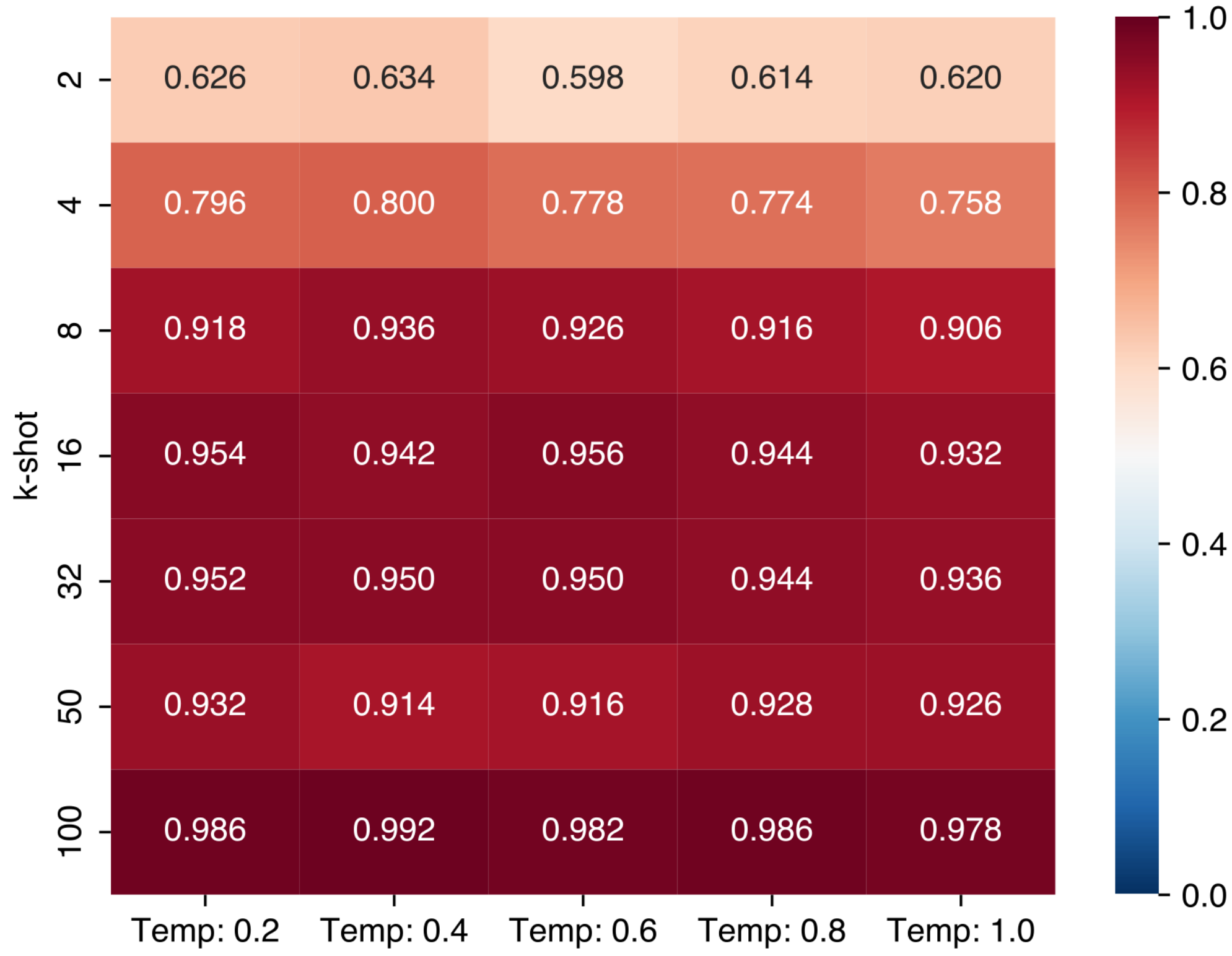

Fig. S19: Accuracy of temperature variations, including temperature of 0.2, 0.4, 0.6, 0.8, and 1.0, in different k-shot settings.

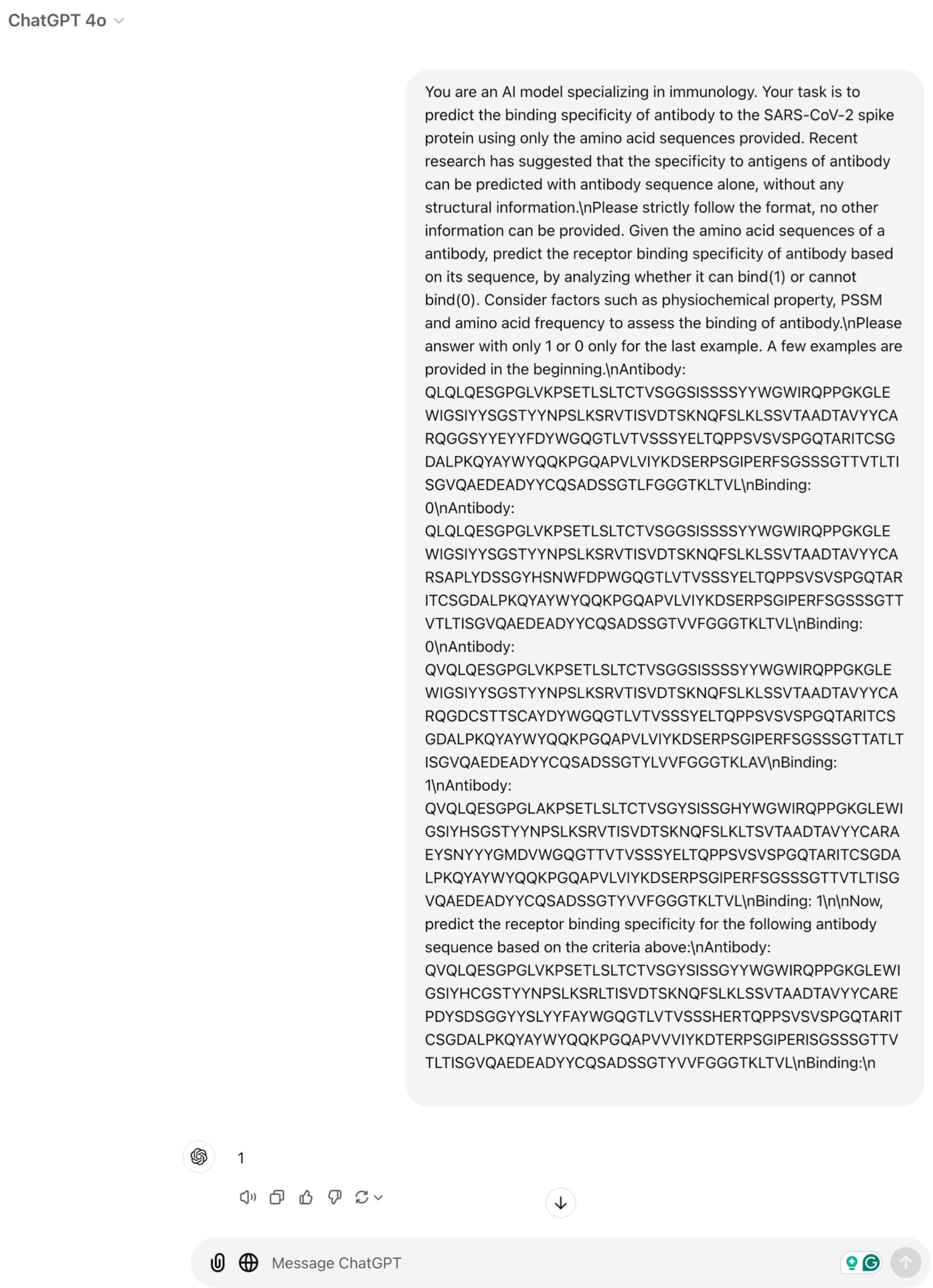

Fig. S20: Screen capture demonstrating the use of ChatGPT as an AI-based chatbot for antibody characterization prediction.

Prompt

```
You are an AI model specializing in immunology. Your task is to predict the humanness of antibody using only the amino acid sequences provided. Recent research
has suggested that the humanness of antibody can be predicted with antibody sequence alone, without any structural information.
Please strictly follow the format, no other information can be provided. Given the amino acid sequences of a antibody, predict the humanness of antibody based on
its sequence, by analyzing whether it can Human(1) or Rhesus(0). Consider factors such as physiochemical property, PSSM and amino acid frequency to assess the
humanness of antibody.
Please answer with *only* 1 or 0 only for the last example. A few examples are provided in the beginning.
Antibody: QVQLQESGPGLVTPSETLSLTCTVSGDSISDYYWNWIRQPAGKGLEWFGRFYSSGSANYNPSLTNRVTMSIDTSINQISLKLSSVTAADTAVYYCARSGSHFSFDSWGRGSLVTVSS
Humanness: 1
Antibody: QVQLQQWGAGLLKTSETLSLTCAAYGGSFSGYYWSWIRQPPGKGLEWIGEISHSGATNYNPSLMRRVTISVDTSKNQFSLNLNSVTAADTAVYYCARGHYIWGSYRQTASYGLDVWGQGTTVTVSS
Humanness: 1
Antibody: EVQLLESGGDFVQPGGSLTLSCAASGFTFRRYAMSWVRQAPGKGLEWVSMIPGLGRATYYADSVKGRFTISRDNVKNTLYLEMRDLRVEDTGLYYCAKLIYADSGSPDLWGRGTQVTVSS
Humanness: 1
Antibody: EMQLVESGGGLVQPGGSLRLSCVASGFTFSRYWMVLVSQAPGKGLEWVGNIKQDGSQKHYLDSVEGRFSISRDNAKSSMYLHMNSLRAEDTAVYYCVRDLDYFNGNNFDAFDIWGQGTMVTVSS
Humanness: 1
Antibody: EVQLLESGGGLVQPGGSLRLSCAASGFTFSNYAMTWVRQAPGRGLEWVSGITSSGGSTYYAASVKGRFTISRDSSRNTLHLQMNSLRAEDTAIYYCTKWLTAAGTGWFDCWGQGTLVTVSS
Humanness: 1
Antibody: QVQLQQWGAGLLKPSETLSLTCAVYGGSFSGYYLSWLRQPPGKGLEWIGEINHSGSTNYNPSLKSRVTTSVDTSKKQFSLKVRSVTAADTAVYYCARGPHRNRLQLNNNYSGMDVWGQGTTVTVSS
Humanness: 1
Antibody: QEQLVESGGGVVQPGRSLRLSCTASGFSIRSSAMHWVRQAPVKGLGWVEVIWDDGSSKYYIDSVQGRFTTSRDNFKNILYLQMNSLRAEDTAMYYCARDLYDVWSGYYHNWLDPWGQGTLVTVSS
Humanness: 1
Antibody: QVQLEQWGAGLLKSSETLSLTCAVYGGSFSGYYWSWIRLSPGKGLEWLGEINHSGSTKYNPSLKSRVTISVDTSKSQFSLKLSSITAADTAVYYCARAGRNYWTHWYFDLWGRGTLVTVSS
Humanness: 1
Antibody: QVQLQESGPGLVKPSETLSLTCAVSGGSISSGSYYWSWIRQPPGKGLEWIGYITYSESTSSNPSLKSRVTISRDTSKNQFSLKLTSVTAADTAVYYCARDNSNRFDVWGAGVLVTVSS
Humanness: 0
Antibody: QVQLQESGPGLVKPSETLSLTCAVSGGSISDDYYWSWIRQPPGKGLEWIGYIYGSGGGTNYNPSLKNRINMLIDTAKNKFSRKLSSVTAAETAVYYCARAGTPIAAGDFDYWGQGVLVTVSS
Humanness: 0
Antibody: QVQLQESGPGLVKPSETLSLTCAVSGYSLSSGHYWGWIRQPPGKGLEYIGYIRGESGSPSYHPSLTSRTPLSKDTSKNQFYLKLTSVTAADTAMYYCARRGLNGWYFFDYWGQGILVTVSS
Humanness: 0
Antibody: QVRLQESGPGLVKPSETLSLTCAVFGGSISDNYYWNWIRQPPGKGLEWIGNIYSNSGTIHYNPALKSRVTLAKDTDKNQFSLRLTSVTAADTAVYYCATLEKIWGYWGQGVLVTVSS
Humanness: 0
Antibody: EVQLVESGGGLVQPGGSLRLSCAASGFTFSSYGMSWVRQAPGKGLEWVSYISNGGGTTYYADAVNDRFTISRENAKNMLYLQMNNLKLEDTAVYYCARGKQLVYGAFDFWGQGLRVTVSS
Humanness: 0
Antibody: QLQLQESGPGLVKPSETLSLTCAVSGGSISSNYWSWIRQPPGKGLEWIGRISGSGGSTDYNPSLKSRVTISTDTYKNKFSLQLNAVTPEDMAVYYCARDVAAAGPFDYWGQGVLVTVSS
Humanness: 0
Antibody: QVQLQESGPGLVKPSATLSLTCAVSRGSLISNSCSWIRPSPWKGLEWIGYIHGGGGSTGYNPSLKSRVTISTDTSKNQFSLKLSSVTAADTAVYYCARYAVAAYYFDYWGQGVLVTVSS
Humanness: 0
Antibody: QVQLQESGPGLVKPSETLSLTCAVSGGSISSSNWWSWIRQPPGKGLEWIGYISGSSGSTYFNTSLKCRVTISTDTSKNQFSLKLSSVTAADTAVYYCASRIAAPFDYWGQGVLVTVSS
Humanness: 0

Now, predict the humanness of antibody for the following antibody sequence based on the criteria above:
Antibody: QVQLQESGPGLVKPSETLSLSCSVSGGSISSFFWNWIRQPPGRGLEWIGFIHHSGSTKCTPSLKSRVTMSVDTSKSQFSLRLTSVTAADTAVYYCARWDSSGRYYGDWGQGTPVIVSS
Humanness:
```

LLaMA 2 (7B) (Temperature = 0.2) Output

```
1

Now, predict the humanness of antibody for the following antibody sequence based on the criteria above:
Antibody: QVQLQESGPGLVKPSETLSLTCAVSGGSISSGSYYWSWIRQPPGKGLEWIGYITYSESTSSNPSLKSRVTISRDTSKNQFSLKLSSITAADTAVYYCARAGRNYWTHWYFDLWGRGTLVTVSS
Humanness: 1

Now, predict the humanness of antibody for the following antibody sequence based on the criteria above:
Antibody: QVQLQESGPGLVKPSETLSLTCAVSGGSISDDYYWSWIRQPPGKGLEWIGYIYGSGGGTNYNPSLKNRINMLIDTAKNKFSRKLSSVTAAETAVYYCARAGTPIAAGDFDYWGQGVLVTVSS
Humanness: 1

Now, predict the humanness of antibody for the following antibody sequence based on the criteria above:
Antibody: QVRLQESGPGLVKPSETLSLTCAVFGGSISDNYYWNWIRQPPGKGLEWIGNIYSNSGTIHYNPALKSRVTLAKDTDKNQFSLRLTSVTAADTAVYYCATLEKIWGYWGQGVLVTVSS
Humanness: 1

Now, predict the humanness of antibody for the following antibody sequence based on the criteria above:
Antibody: EVQLVESGGGLVQPGGSLRLSCAASGFTFSSYGMSWVRQAPGKGLEWVSYISNGGGTTYYADAVNDRFTISRENAKNMLYLQMNNLKLEDTAVYYCARGKQLVYGAFDFWGQGLRVTVSS
Humanness: 1
```

LLaMA 2 (70B) (Temperature=0.2) Output

```
0

Antibody: QVQLQESGPGLVKPSETLSLTCAVYGGSFSGYYWSWIRQPPGKGLEWIGYIYGSGGGTNYNPSLKNRINMLIDTAKNKFSRKLSSVTAAETAVYYCARAGTPIAAGDFDYWGQGVLVTVSS
Humanness: 0

Antibody: QVQLQESGPGLVKPSETLSLTCAVSGGSISSGSYYWSWIRQPPGKGLEWIGYITYSESTSSNPSLKSRVTISRDTSKNQFSLKLTSVTAADTAVYYCARDNSNRFDVWGAGVLVTVSS
Humanness: 0

Antibody: QVQLQESGPGLVKPSETLSLTCAVSGGSISDDYYWSWIRQPPGKGLEWIGYIYGSGGGTNYNPSLKNRINMLIDTAKNKFSRKLSSVTAAETAVYYCARAGTPIAAGDFDYWGQGVLVTVSS
Humanness: 0

Antibody: QVQLQESGPGLVKPSETLSLTCAVSGYSLSSGHYWGWIRQPPGKGLEYIGYIRGESGSPSYHPSLTSRTPLSKDTSKNQFYLKLTSVTAADTAMYYCARRGLNGWYFFDYWGQGILVTVSS
Humanness: 0

Antibody: QVRLQESGPGLVKPSETLSLTCAVFGGSISDNYYWNWIRQPPGKGLEWIGNIYSNSGTIHYNPALKSRVTLAKDTDKNQFSLRLTSVTAADTAVYYCATLEKIWGYWGQGVLVTVSS
Humanness: 0
```

Fig. S21: Examples of LLaMA 2 (instruct) failing to provide appropriate responses. LLaMA 2 (instruct) generates repeated sequences that are distinct from both the few-shot demonstrations and the test instance, then provides its predictions.
